# Improving genomic prediction accuracy for methane emission and feed efficiency in sheep: integrating rumen microbial PCA with host genomic variation using neural network GBLUP (NN-GBLUP)

**DOI:** 10.1186/s12711-025-00987-x

**Published:** 2025-07-17

**Authors:** Setegn Worku Alemu, Timothy P. Bilton, Patricia L. Johnson, Benjamin J. Perry, Hannah Henry, Ken G. Dodds, John C. McEwan, Suzanne J. Rowe

**Affiliations:** AgResearch, Invermay Agricultural Centre, Private Bag 50034, Mosgiel, 9053 New Zealand

## Abstract

**Background:**

Methane emissions from ruminant livestock pose a significant challenge to mitigating climate change. Genomic selection offers a promising approach to reduce methane emissions, but prediction accuracy remains low due to the high cost of measuring methane emissions. Integrating rumen microbiome composition (RMC) data may improve genomic prediction accuracy, yet the high dimensionality of RMC data presents computational challenges. This study aimed to (1) evaluate the effectiveness of principal component analysis (PCA) for reducing RMC data dimensionality while retaining essential information, and (2) assess whether incorporating PCA-reduced RMC data as intermediate traits in a Neural Network Genomic Best Linear Unbiased Prediction (NN-GBLUP) model improves genomic prediction accuracy for methane emissions and feed efficiency traits in sheep.

**Results:**

For the first objective, Principal Components (PCs) explaining 100% of variation effectively captured RMC information, with microbiability estimates closely matching those from the full dataset. For the second objective, the NN-GBLUP model incorporating PCA-reduced RMC data improved prediction accuracy compared to standard GBLUP methods. Prediction accuracy for methane emissions increased from 0.09 to 0.30 in train-test validation and from 0.15 to 0.27 in five-fold cross-validation using PCA components explaining 25% of total RMC variation. For residual feed intake, accuracy improved from 0.25 to 0.37 in train-test validation and from 0.25 to 0.34 in cross-validation. Optimal PCA components varied by trait, with 25% and 50% components showing the best results. Prediction accuracy did not improve for carbon dioxide emissions, live weight, and mid-intake, indicating trait-dependent microbiome influence.

**Conclusions:**

Principal Component Analysis reduced the dimensionality of rumen microbiome data while preserving essential biological information. The integration of these PCA-reduced data with host genomic information through an NN-GBLUP model substantially improved genomic prediction accuracy for methane emissions and feed efficiency in sheep. Principal components explaining 25% and 50% of the variation yielded the highest accuracy, whereas higher components (75% and 95%) reduced accuracy for methane traits. This approach shows promise for implementing genomic selection strategies to reduce methane emissions and improve feed efficiency in ruminant livestock in a computationally efficient manner, thereby contributing to climate change mitigation efforts in agriculture.

**Supplementary Information:**

The online version contains supplementary material available at 10.1186/s12711-025-00987-x.

## Background

Enteric methane emissions from agricultural ruminants pose a significant challenge to mitigating anthropogenic global warming. In parallel, the livestock industry has been focusing on improving feed efficiency, where one commonly derived trait is residual feed intake (RFI). Methane emissions and RFI are closely interconnected traits of primary importance in ruminant livestock production as: methane emissions have significant environmental impact, while RFI directly affects economic efficiency. Enteric methane is a byproduct of a symbiotic relationship with microorganisms in the rumen, which convert forage into a mixture of volatile fatty acids and protein [1, 2]. However, this symbiosis also produces methane, a potent greenhouse gas, during enteric fermentation in the rumen [3]. With ruminants contributing approximately 40% of global methane emissions [4], and with atmospheric methane reaching unprecedented levels, the need for effective mitigation strategies has become urgent. Hence, the United Nations set a target in 2020 to reduce methane emissions 30% by 2030. There is growing interest in exploring potential connections between feed efficiency and environmental impacts, due to the complex interactions between animal nutrition, metabolism, and greenhouse gas production in ruminants. RFI represents the difference between an animal's actual feed intake and its expected feed requirements for maintenance and production. From an economic perspective, RFI is an important trait due to its impact on production costs.

Traditional approaches to reducing methane emissions in ruminants, such as dietary changes and feed additives, have shown potential [5]. However, these methods face challenges in consistent implementation and in addressing the adaptability of rumen microbiomes [5–7]. As an alternative to conventional methods, genetic selection presents a sustainable, long-term approach to reducing methane emissions and improving feed efficiency in ruminants [8–11]. By leveraging the heritable nature of these traits, a gradual decrease in emissions across generations is possible [8–10]. However, the low to moderate heritability of methane traits and RFI, ranging from 0.16 to 0.25 [12–14] and 0.11 to 0.3 [13, 15, 16], respectively; and the difficulty in obtaining accurate phenotypic measures of methane emissions and feed efficiency on a large scale, pose major challenges to their use in commercial breeding programs. Moreover, genomic prediction accuracy for methane remains limited, largely because of the limited number of measurements available; for example, Hess et al. [13] reported an accuracy of 0.13 for methane and 0.29 for RFI.

One strategy to increase the accuracy of genomic selection involves expanding the reference population, i.e., increasing the number of genotyped and phenotyped individuals [17]. However, recording large numbers of individuals for methane traits and RFI is costly. An alternative strategy involves selecting for correlated traits that exhibit higher heritability. However, this approach may not be optimal for methane emissions, as production-related traits demonstrate low genetic correlations with methane [18, 19]. Another potential solution might be to incorporate rumen microbiome composition (RMC), information where it is possible to collect many samples from industry animals. The rumen microbiome, composed of microorganisms that partially ferment the ingested feed, plays a crucial role in digestion and affects methane emissions [20] and feed efficiency [21–23]. These microbes influence methane emissions primarily through methanogenesis, a process carried out by methanogenic archaea. In addition to its impact on methane emissions, the rumen microbiome has also been associated with feed efficiency [21–24]. Both methane emissions and feed efficiency-related RMC are heritable [13, 22]. For example, Wallace et al. [25] reported a heritability estimate greater than 0.2 for methane emissions, while Li et al. [22] found a heritability of over 0.15 for feed efficiency, suggesting that these traits can be targeted through genetic selection.

Given the heritable nature of RMC, incorporating RMC information into genomic selection models may improve the accuracy of genomic predictions for these traits. Recent advancements in high-throughput sequencing technologies have increased the availability of RMC data, facilitating this integration. However, determining the optimal approach for incorporating RMC information remains an active area of research. Traditionally, RMC information has been fitted as an additional non-genetic random term in models [13, 20, 26]. This approach allows for the simultaneous estimation of additive genetic variation and microbial variation. The contribution of RMC to phenotypic variation, a concept known as 'Microbiability,' highlights the substantial influence of rumen microbes on traits such as feed efficiency and methane emissions [20]. Despite the insights gained from this traditional approach, there may be more effective methods for integrating RMC information. The heritable nature of RMC and their partial control by the host suggest that treating rumen microbiome data merely as an additional non-genetic random term may not be optimal as this assumes that the covariance between the genetic and rumen microbiome terms are zero, which is known to be false. A more optimal strategy that captures the heritable portion of the rumen microbiome variation could potentially improve the accuracy of genomic selection for methane emissions and feed efficiency in ruminants.

Several alternative methods for integrating RMC information into genetic evaluations have been proposed. One approach involves integrating rumen microbiome-derived phenotypes as correlated traits in multi-trait genetic models, similar to how Hayes et al. [27] used Near-Infrared Spectroscopy and Nuclear Magnetic Resonance predictions as correlated traits to improve genomic prediction accuracy, although this might not be optimal due to the high dimensionality of the resultant multi-trait prediction [28]. Another approach might be incorporating RMC information in a two-step approach [29], where breeding values are considered both through direct genetic effects and through microbiota-mediated pathways. Christensen et al. [28] proposed a joint model that accounts for genetic effects mediated by intermediate traits, such as RNA, which directly link DNA action to phenotype expression. While Christensen’s work focused on RNA as an intermediate trait, the underlying principle—using intermediate layers to capture genetic effects—can be extended to other omics data, including the rumen microbiome. Zhao et al. [30] expanded the methodology of Christensen et al. [28] by introducing the Neural Network-Mixed Model (NN-MM), a Bayesian framework allowing for different heritabilities for each omics feature. This method might be an optimal solution for integrating rumen microbiome data. While the rumen microbiome is not a direct intermediate trait of the host (like RNA is an intermediate trait between DNA and phenotype), it is directly involved in enteric-methane metabolism and fitting it as an intermediate layer in methane trait prediction is cogent [31]. As such, although RMC data are not strictly intermediate traits, they serve as a source of information between genotype and phenotype.

However, the implementation of such models, particularly when dealing with high-dimensional rumen microbiome data, still presents significant challenges. In NN-MM, the intermediate layer would require as many nodes as the dimension of the omics data. For instance, if the RMC data contains 200,000 features, this would necessitate running 200,000 individual genomic prediction models (either genomic best linear unbiased prediction (GBLUP) or Bayesian models, depending on the specific NN-MM architecture), which is computationally prohibitive. This high dimensionality increases computational complexity and raises concerns about overfitting and the 'curse of dimensionality', potentially compromising the model's predictive power and generalizability. To overcome these challenges while still leveraging the benefits of rumen microbiome data, a solution is needed that can reduce the dimensionality of the RMC data without significant loss of information. We propose an integrated methodology that combines dimensionality reduction techniques with NN-MM, specifically an implementation of the NN-MM method known as Neural Network GBLUP (NN-GBLUP). This approach aims to address the high-dimensionality issue while retaining the benefits of treating RMC data as an intermediate layer.

Our study has two main objectives:

1. Preprocessing high-dimensional RMC data using Principal Component Analysis (PCA) and validating its effectiveness by comparing microbiability estimates from PCA subsets to full dataset estimates.
2. Evaluating genomic prediction accuracy by testing whether integrating reduced-dimensional RMC information as an intermediate trait improves predictions for traits influenced by RMC.

We compare the NN-GBLUP against two alternatives: GBLUP model and a model that extends the GBLUP approach by incorporating rumen microbiome data as an additional, independent random effect alongside the genomic information. We employ both year-based train-test splits and cohort-based five-fold cross-validation to address potential dispersion bias from correlated rumen microbiomes within cohorts.

## Methods

### Ethical considerations

The animal experiments conducted adhered to the guidelines of the 1999 New Zealand Animal Welfare Act and AgResearch Code of Ethical Conduct. The trials of the current study were approved by the AgResearch Invermay (Mosgiel, NZ) Animal Ethics committee with the approval numbers: 13081, 13419, 13563, 13742, 13892, 14055, 14066, and 14221.

### Data description

The dataset employed in this analysis comprised of the samples from the grass lamb and lucerne lamb groups from Hess et al. [13]. It consisted of 1051 animals in the Methane Grass (i.e., grass lamb) group and 984 animals in the RFI Lucerne (i.e., lucerne lamb) group, born between 2014 and 2016, where 863 animals were included in both groups. Animals were from one of three New Zealand flocks and were measured as lambs (< 15 months). Methane phenotypes were measured using portable accumulation chambers (PAC) for the Methane Grass group, and feed intake traits were measured in the feed intake facility at AgResearch's Invermay campus for the RFI Lucerne group as described in Hess et al. [13]. For the Methane Grass group, the PAC traits measured was scaled by dividing by the lot mean and multiplying by the population mean similar approach as Jonker et al. [12] and analysis performed using scaled methane emissions (CH4, g/day), scaled methane ratio (CH4Ratio), calculated as CH4/(CO2 + CH4) and expressed as mM/Mol), liveweight at the time of rumen sample collection (LW, kg), and scaled carbon dioxide emissions (CO_2_, g/day). For the RFI Lucerne group, animals were transitioned to a lucerne pellet diet over a 2-week period. Following this, daily intake data was collected for approximately 42 consecutive days. Derived traits for analysis were mid-trial intake and residual feed intake (RFI). Mid-trial intake was computed for each animal as the predicted mean (at day 21) from a linear regression model of the animal’s daily intakes, and RFI was calculated as the residual of a model where intake was fitted as the dependent variable, with metabolic weight, growth rate, cohort, flock, and pen fitted as explanatory variables.

### Genotypes and SNP selection

All individuals were genotyped using SNP chips of varying densities and imputed to 600 K SNPs (high-density) as described in Hess et al. [13]. Quality control measures were applied, which included filtering SNPs based on unique mapping to the Ovis aries (OAR) v3.1 genome reference and other criteria [13]. For our prediction modelling, we started with this imputed 600 K SNP set. To address computational constraints associated with Bayesian model computation, we created two subset panels: a 50 K SNP set and a 15 K SNP set. These subsets were created by selecting SNPs on the OvineSNP50 panel and on the ISGC_SheepLD15K panel (https://figshare.com/articles/dataset/Mapping_of_ISGC_SNP_chip_probes/8424935 accessed 2/Sep/2024), respectively. Preliminary analyses showed similar genomic prediction accuracy across the 15 K, 50 K, and 600 K SNP sets (results not shown). Consequently, for this study, we focused on using the 15 K genotype data for our main analyses. The total number of quality control SNPs for the 15 K genotype used in this study was 11,967.

### Rumen microbiome sampling and sequencing

Rumen microbiome samples were collected at the time of PAC measurements for the animals in the Methane Grass group and during the feed intake trial for the animals in the RFI Lucerne group, and microbiome sequencing was performed using restriction enzyme-reduced representation sequencing [32]. Rumen contents were sampled via stomach intubation, freeze-dried, and DNA was extracted using a combined beat-beating, phenol and column purification protocol. Rumen microbiome composition (RMC) profiles were generated from the sequence data using a reference-free pipeline developed by Hess et al. [32]. The reference-free approach was chosen as it demonstrated substantially higher prediction accuracies compared to reference-based methods. For example, Hess et al. [13] found that the prediction accuracy from using the reference-free approach improved twofold compared to using a reference-based approach. The reference-free pipeline generates a RMC profile consisting of counts of unique 65 bp microbial sequences, referred to as a tag, that are present in 25% of the samples (i.e., at least one read) and resulted in 240,743 microbial tags for the Methane Grass group and 207,393 microbial tags for the RFI Lucerne group. The counts of these tags were computed to generate the rumen microbiome profiles, and were pre-processed using the approach described in Hess et al. [13] that consisted of the following steps: (a) counts were transformed by adding one to each count, dividing by the sum of total reads and columns, and then applying a log10 conversion; (b) the transformed count data were normalized by scaling each column to mean 0 and variance 1 within each rumen cohort, where a rumen cohort was defined as the set of animals from the same flock and birth year measured over the same ~ 2 day period, of the same sex, and sequenced on the same sequencing flow cell.

### Validation

For both the Methane Grass and RFI Lucerne groups, we employed two prediction approaches: a train-test split based on year and a five-fold cross-validation based on cohort. In the train-test split, we trained the model using data from 2014 to 2015 (690 individuals for Methane Grass, 587 for RFI Lucerne) and tested it on the 2016 data (361 and 397 individuals, respectively). This temporal division allowed us to assess the model's generalizability across different cohorts and environmental conditions. For the cohort-based cross-validation, each cohort was divided into five sub-groups by randomly assigning animals within each cohort to ensure approximately equal sub-group sizes. For example, in a cohort of 10 animals, random assignment would result in five sub-groups of two animals each. In each validation fold, one sub-group from every cohort served as part of the validation dataset, while the remaining four sub-groups from all cohorts formed the training set. This process was repeated five times, with each sub-group serving as part of the validation set exactly once. This approach resulted in validation set sizes of 214, 208, 203, 208, and 218 animals for Methane Grass, and 199, 196, 195, 196, and 198 animals for RFI Lucerne.

### Statistical method

#### Fixed effects and phenotype adjustment

Raw phenotypes were adjusted for fixed effects that consisted of the fixed class for birth and rearing rank, the fixed class for age of dam (1, 2, 3 +), contemporary group which is the combination of flock and birth year, and the covariate of birthday deviation from the mean birthdate of all animals born from the same flock and birth year. The adjustment of phenotypes involved the following equation:

1 $${\mathbf{y}} = {\mathbf{X\beta }} + {\mathbf{e}},$$

where $\mathbf{y}$ represents a vector of phenotypes and ${\varvec{\upbeta}}$ represents the fixed class and covariate effects listed above. By employing this equation, the residuals, or adjusted phenotypes ***(***$\mathbf{y}-\mathbf{X}\widehat{{\varvec{\upbeta}}}$***)*** were obtained and subsequently utilized for the genomic evaluation.

#### Preprocessing of high-dimensional microbiome data

To manage the "small n, large p" challenge prevalent in preprocessing high-dimensional microbial data, we implemented PCA using R [33]. The primary goal was to minimize data dimensionality while maximizing the retention of original variance. Despite some similarities in data between the two groups, PCA was performed individually for both the Methane Grass group and the RFI Lucerne group to better accommodate their unique RMC. For each group, PCA was performed on the RMC from the combined train and test populations. As the response variable is not involved in the PCA process, no information about the trait of interest from the test set was used in the training.

**Fig. 1:**
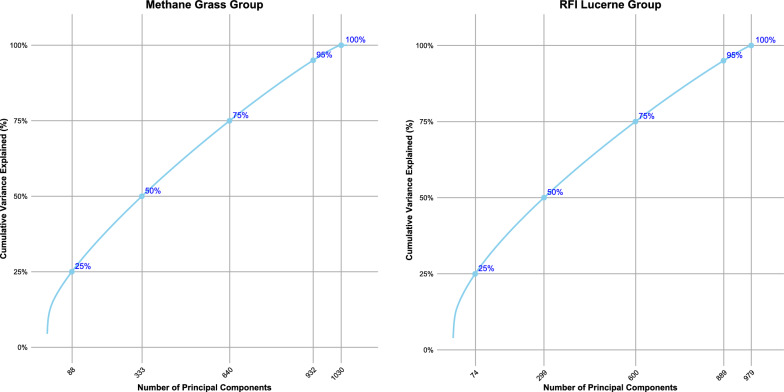
Principal component analysis of standardized microbial tag counts. **a** Methane Grass group: methane, methane ratio, carbon dioxide, and live weight **b** RFI Lucerne group: RFI (Residual Feed Intake) and mid intake traits

To assess the effectiveness of variance reduction, we generated scree plots for each group. These plots illustrated the cumulative variance explained as a function of the number of principal components (PC) retained (Fig. 1). Markers were placed on the scree plots at the following variance thresholds: 25%, 50%, 75%, 95%, and 100% to determine the minimal number of components necessary for capturing a substantial portion of the data’s variance.

Identifying these specific variance thresholds is essential for our subsequent analyses, especially considering the computational demands of the Bayesian neural network model.

#### Microbiability estimation

To address the first objective of our study, we conducted microbiability estimation to validate the effectiveness of the PCA-based dimension reduction. Microbiability quantifies the rumen microbiome influence on specific phenotypes, such as methane and RFI traits. The effectiveness of the dimension reduction was assessed by comparing the microbiability estimate derived from PC explaining 100% of the variation with the estimate obtained using the full rumen microbiome dataset. A close alignment between these estimates indicates successful dimension reduction, suggesting that the PCA approach effectively captures the essential rumen microbiome information.

The following model was employed for microbiability estimation:

2 $${\varvec{y}}_{{{\varvec{adj}}}} = 1{\varvec{\mu}} + {\varvec{Z}}_{{\varvec{m}}} {\mathbf{m}} + {\mathbf{e}},$$

where ${{\varvec{y}}}_{{\varvec{a}}\mathbf{d}\mathbf{j}}$ is a vector of adjusted phenotype for the trait (methane, methane ratio etc.), $\mathbf{1}$ is the vector of 1’s,  $\upmu$ is the population mean, ${{\varvec{Z}}}_{\mathbf{m}}$ is an incidence matrix for the rumen microbiome effect, m is the vector representing rumen microbiome effects, assumed to follow a normal distribution:, $\mathbf{m} \sim \mathbf{N}\left(0,\mathbf{M}{\sigma }_{m}^{2}\right)$, M is the rumen microbiome similarity matrix, computed as the correlation between scaled rumen microbial tag counts, ${\sigma }_{m}^{2}$ is the microbiome effect variance, and $\mathbf{e}$ is a vector of residuals assumed to follow a normal distribution $\mathbf{e} \sim \mathbf{N}\left(\mathbf{0},{\sigma }_{e}^{2}\right)$ where ${\sigma }_{e}^{2}$ is the residual variance. Microbiability was calculated as ${\sigma }_{m}^{2}/({\sigma }_{m}^{2}+{\sigma }_{e}^{2})$. We used ASReml v4.2 [34] to estimate the microbiability.

#### Genomic prediction methods

The following three methods were employed for genomic prediction and their accuracies were compared, addressing objective two of our study.

***Method 1: Genomic Best Linear Unbiased Prediction (GBLUP)***

The first method employed was GBLUP, with the following model:

3 $${\mathbf{y}}_{{{\mathbf{adj}}}} = {\mathbf{1}}{\varvec{\mu}} + {\mathbf{Z}}_{{\varvec{u}}} {\mathbf{u}} + {\mathbf{e}},$$

where $${\mathbf{y}}_{\mathbf{a}\mathbf{d}\mathbf{j}}$$$$\mathbf{1}$$, $${\mu}$$, and $$\mathbf{e}$$ were defined earlier, $${\mathbf{Z}}_{{\varvec{u}}}$$ is an incidence matrix for the additive genetic effect, $${\varvec{u}}$$ is a vector of breeding value assumed to follow a normal distribution$$\mathbf{u}\boldsymbol{ }\sim \mathbf{N}\left(\mathbf{0},\mathbf{G}{\sigma }_{u}^{2}\right)$$, where $$\mathbf{G}$$ is a genomic relationship matrix computed based on the first method proposed by VanRaden [35] and $${\sigma }_{u}^{2}$$ represents the additive genetic variance.

***Method 2: GBLUP with Microbiome as Extra Non-Genetic Random Term (GM)***

The second method extended the GBLUP model by incorporating the microbiome as an extra non-genetic random term:

4 $${\mathbf{y}}_{{{\mathbf{adj}}}} = {\mathbf{1\mu }} + {\mathbf{Z}}_{{\varvec{u}}} {\mathbf{u}} + {\mathbf{Z}}_{{\varvec{m}}} {\mathbf{m}} + {\varvec{e}},$$

where ${\mathbf{y}}_{\mathbf{a}\mathbf{d}\mathbf{j}}$, $\mathbf{1}, {\mu}, {{\varvec{Z}}}_{{\varvec{u}}}$, $\mathbf{u}$, ${{\varvec{Z}}}_{{\varvec{m}}}$, $\mathbf{m}$, and $\mathbf{e}$ were defined as before. It is important to note that in this method, the $\mathbf{m}$ matrix is based on the full rumen microbial tags, not the PCA-reduced data. This approach allows for the inclusion of all available rumen microbiome information without dimensionality reduction. For the estimation of microbiability and the implementation of Methods 1 and 2 (GBLUP and GBLUP with rumen microbiome effects), we used ASReml v4.2 [34].

***Method 3: Neural Network Linear Mixed Model (NN-MM) with NN-GBLUP implementation***

The third method employed a multilayer neural network linear mixed model (NN-MM) developed by Zhao et al. [30] to integrate genomic variations with phenotypic traits, while considering the linear interactions captured by the principal component analysis (PCA) of the rumen microbiome data. In this study, we used an implementation of NN-MM, known as Neural Network GBLUP (NN-GBLUP), which incorporates GBLUP into a neural network framework, assuming a normal distribution with equal variance for all marker effects. In this study, we implemented the NN-GBLUP model using JWAS (Julia Whole-genome Analysis Software) [36]. As described by Zhao et al. [30], the NN-MM model architecture comprises three distinct layers. The first is an input layer containing genotypes. The second layer is an intermediate layer that serves as an observable bridge between the genotype data and the phenotypic traits, capturing the linear relationships between them. In this study, the intermediate layer represents the rumen microbiome information through PCA components, which are derived from the high-dimensional RMC data. This approach allows us to effectively reduce the dimensionality of the rumen microbiome data while retaining its most important features. The last layer is the output layer that represents the phenotypic traits of interest, such as methane emissions, methane ratio, or other relevant traits.

*Transition from input layer to intermediate layer*

For the transition from the input layer, consisting of genotypes, to the intermediate layer, encapsulating microbiome PCA data, we adopted a mixed model approach. This approach can be represented as follows:

$$pc_{ij} = \mu_{j}^{\left( 0 \right)} + \mathop \sum \limits_{k = 1}^{K} x_{i,k} w_{j,k}^{\left( 0 \right)} + \varepsilon_{ij.}$$

Here ${pc}_{ij}$ denotes the ${j}^{th}$ intermediate rumen microbiome PC feature ($j=\text{1,2},\dots ,J$ where $J$ is the total number of rumen microbiome PCA components used), ${x}_{i,k}$ is the genotype covariate at locus $k$ ($k=\text{1,2},\dots ,K)$ for individual$i$, ${w}_{j,k}^{\left(0\right)}$ represents the effect of locus $k$ on the $j$^*th*^ PC feature (intermediate rumen microbiome PC feature which is the weight between the ${k}^{th}$ node of the input layer and ${j}^{th}$ node intermediate rumen microbiome layer) and ${\varepsilon }_{ij}$ is the random residual for the ${j}^{th}$ PC feature. Under the GBLUP framework, ${w}_{j,k}^{\left(0\right)}$ is assumed to follow a normal distribution with mean zero and variance ${\varvec{G}}{\upsigma }_{{{j}^{w}}^{\left(0\right)}}^{2}$, where the genomic relationship matrix, G = XX'/K where X is the centered and scaled genotype matrix and K is the number of SNP. A separate GBLUP model was fitted for each rumen microbial PC. For example, for PC 88, 88 GBLUP models was fitted. A flat prior was applied to the overall mean, ${\mu }_{j}^{(0)}$ and the random residuals for each principal componenets feature, ${\varepsilon }_{ij}$, was assumed to have independently and identically distributed normal priors with null mean and variance ${\sigma }_{{\varepsilon }_{j}}^{2}$. The variance parameters ${\sigma }_{{{j}^{w}}^{\left(0\right)}}^{2}$ and ${\sigma }_{{\varepsilon }_{j}}^{2}$ were assumed to follow scaled inverse chi-squared distributions.

The relationship between the input and intermediate layers was modelled using multiple independent GBLUP analyses. Specifically, a separate GBLUP model was fitted for each rumen microbiome PC component, treating each PC as an independent trait.

*Transition from intermediate layer to output layer*

Transitioning from the intermediate layer to the output layer, which represents the phenotype, is modelled as follows:

$$y_{i} = \mu^{\left( 1 \right)} + \mathop \sum \limits_{j = 1}^{J} w_{j}^{\left( 1 \right)} I(pc_{ij} ) + e_{i} .$$

In this model, ${\text{y}}_{\text{i}}$ is the phenotype for individual $i$, ${\mu }^{\left(1\right)}$ is the overall mean, ${pc}_{ij}$ is the jth rumen microbiome PC feature of individual $i$, $I\left(.\right)$ is the activation function within the neural network (a linear function was utilized), ${w}_{j}^{\left(1\right)}$ is the effect of ${I(z}_{i,j})$ on ${y}_{i}$, ${e}_{i}$ is the random residual. The priors for the neural network weights ${w}_{j}^{\left(1\right)}$ and residuals ${e}_{i}$ are normally distributed with zero mean and variances ${\sigma }_{{w}^{\left(1\right)}}^{2}$ and ${\sigma }_{\epsilon }^{2}$ respectively. Both ${\sigma }_{{w}^{\left(1\right)}}^{2}$ and ${\sigma }_{\varepsilon }^{2}$ are presumed to follow a scaled inverse chi-square distribution. Our analysis involved 60,000 chains with a burn-in phase of 10,000, sampled every 10 steps.

Prediction of genomic breeding value: In our implementation of the NN-MM model with NN-GBLUP, we used a linear activation function and had no missing omics data. Under these conditions, the calculation of breeding values can be described as follows:

$${GEBV}_{i}=X\widehat{{W}^{\left(0\right)}}{w}^{\left(1\right)}$$

where, $\text{X}$ is the genotype matrix, $\widehat{{\text{W}}^{\left(0\right)}}$ is the matrix of estimated marker effects on the intermediate omics features (PCA components of microbiome data),${\text{w}}^{\left(1\right)}$ is the vector of estimated effects of the intermediate omics features on the phenotype.

One question that arises when using PCs as intermediate traits representing microbiome effects is how many PCs should be fitted. This is a meta-parameter that needs to be determined using cross-validation, fitting models with varying numbers of PCs. Given computational constraints, we selected PCs explaining different proportions of the total variation in the rumen microbiome data for both the RFI Lucerne and Methane Grass groups. For the train-test cross-validation, PCs explaining 25%, 50%, 75%, and 95% of the variance were chosen. In contrast, for the five-fold cross-validation, we selected PCs that explained only 25%, 50%, and 75% of the variance. It is important to note that we did not implement five-fold cross-validation using PCs that explained 95% of variation, to concentrate computational effort on the more promising scenarios. Models were then fitted using these different subsets of PCs, and their performance was evaluated using cross-validation. The optimal number of PCs for each trait and population was selected based on the model's performance, considering the trade-off between the number of components, the explained variation, and the model's predictive ability.

### Models evaluation

To evaluate the performance of the three models, we employed two metrics: genomic prediction accuracy and dispersion bias. These metrics were calculated using the test population data, where genotype and rumen microbiome information were available, but phenotype data was set to missing. Genomic prediction accuracy was computed as the correlation between adjusted phenotypes and genomic estimated breeding values. Dispersion bias was computed as the regression slope coefficient of adjusted phenotypes on genomic estimated breeding values. For the train-test cross-validation, the standard error for bias was computed by fitting a linear model where GEBVs were the response variable and adjusted phenotypes were the independent variable. The standard error of the slope was used to estimate the standard error of the bias.

The standard error of the genomic prediction accuracy was computed using the formula:$\frac{1-{\widehat{{\varvec{r}}}}^{2}}{\sqrt{n}}$ where *n* refers the number of individuals to the validation population ${\widehat{r}}^{2}$ the estimated squared correlation between the adjusted phenotype and estimated breeding value [37]. For the five-fold cross-validation, the standard error of the dispersion bias and genomic prediction accuracy was computed by taking the standard deviation of the dispersion bias and genomic prediction accuracy across the five folds.

## Results

In this study, we addressed two main objectives: first, to evaluate the effectiveness of Principal Component Analysis (PCA) in preprocessing high-dimensional rumen microbiome composition (RMC) data, and second, to assess the impact of integrating this RMC information on genomic prediction accuracy for six traits in ruminants.

**Fig. 2:**
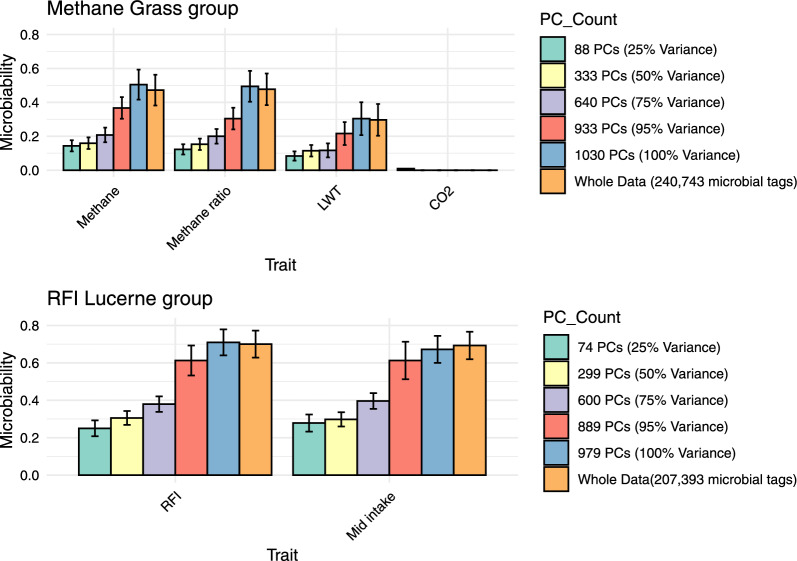
Microbiability estimates across PCA component thresholds and whole data (full microbial tags). **a** Methane Grass group: methane, methane ratio, LWT, and CO_2_. **b** RFI Lucerne group: RFI, mid intake

The estimated microbiabilities for all traits using various numbers of PCA components that explained 25%, 50%, 75%, 95%, and 100% of the variance in the RMC data are shown in Fig. 2 along with the estimates obtained from using all rumen microbial tags. We observed a consistent pattern across all traits: as the number of PCA components increased, explaining a higher percentage of variance, the microbiability estimates also increased. Specifically, for the Methane Grass group, microbiability estimates for methane and methane ratio traits showed a consistent increase from 25% variance explained (88 PCA components) to 100% variance explained (1030 PCA components) as shown in Fig. 2. At 100% variance explained, microbiability from the PCA estimates closely matched those from the full dataset (all rumen microbial tags) estimate. For methane, the PCA estimate was 0.50 compared to 0.47 using the full dataset, while for methane ratio, the PCA estimate was 0.49 versus 0.48 for the full dataset. Live weight (LWT) exhibited a similar trend, with an estimate of microbiability of 0.30 using PCs explaining 100% variance, closely aligning with the full dataset estimate of 0.30. Both PCA and full dataset approaches yielded zero microbiability estimates for CO_2_ emissions. In the RFI Lucerne group, microbiability estimates for RFI and mid intake traits increased from 25% variance explained (74 PCA components) to 100% variance explained (979 PCA components). At 100% variance explained, the PCA estimate for RFI was 0.71 compared to 0.70 for the full dataset, while for mid intake, the PCA estimate was 0.67 versus 0.69 for the full dataset.

For the second objective, we evaluated three models: two baseline Genomic Best Linear Unbiased Prediction (GBLUP) models and one Neural Network GBLUP (NN-GBLUP) model. The first baseline model (Model 1, referred to as G) did not include rumen microbial count (RMC) data, while the second baseline model (Model 2, referred to as GM) incorporated full rumen microbial tag count data as an additional non-genetic term. The third model (Model 3) was an NN-GBLUP model that integrated PCA-reduced RMC information.

To further investigate the impact of dimensionality reduction on Model 2 (GM), we tested multiple GM models with varying proportions of PCA-reduced microbiome data, explaining 25%, 50%, 75%, and 95% of the total variation. These were compared against the GM model using the full microbial count data. The results revealed that the GM models with 50%, 75%, and 95% of the variation explained by PCA achieved prediction accuracies comparable to the GM model using the full microbial count, while the 25% PCA-reduced model exhibited slightly lower accuracy. Detailed prediction accuracies for the GM models with different PCA-reduced proportions are provided (see Additional file 3, Figure S1, Additional file 4, Figure S2, Additional file 5, Figure S3 and Additional file 6, Figure S4).. Based on these findings, we selected the GM model using the full microbial count as the representative of the GM framework for comparison with Model 1 (G) and Model 3 (NN-GBLUP).

The genomic prediction accuracies of Model 1 (G) and Model 2 (GM) were similar. This similarity, despite high microbiability estimates, can be attributed to the statistical treatment of the microbiome information in Model 2. Specifically, the microbiome data was fitted as an independent random term, assuming zero covariance between microbiome and genomic information. While computationally convenient, this assumption of independence does not leverage potential interactions between host genetics and microbiome composition, thereby limiting improvements in genomic prediction accuracy.

We chose Model 2 (GM) as the baseline for comparison with Model 3 (NN-GBLUP) because, unlike Model 1 (G), Model 2 incorporates rumen microbiome data, making it a more suitable candidate for evaluating the added value of integrating microbiome information in different ways. Both Model 2 and Model 3 incorporate similar information on genomics and rumen microbes but differ in their integration methods. This comparison allows us to assess whether incorporating RMC information as an observed intermediate trait in the NN-GBLUP model (Model 3) provides advantages over treating rumen microbiome data as an independent non-genetic term in the standard GBLUP model (Model 2). To evaluate model performance, we conducted both train-test validation and five-fold cross-validation.

### Train-test validation results

#### Methane Grass group

**Fig. 3:**
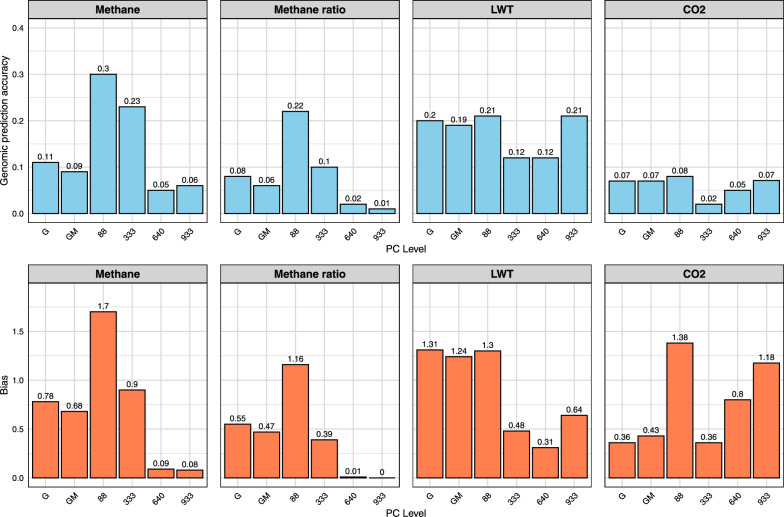
Genomic prediction accuracy and dispersion bias for methane Grass group (2016 Testing). Traits: methane, methane ratio, LWT, CO_2_. Methods: G (GBLUP), GM (Method 2), NN-GBLUP (88, 333, 640, 933 PCs)

As can be seen in Fig. 3, the NN-GBLUP model incorporating PCA components explaining 25% of the rumen microbiome variation (88 PCs) substantially improved the genomic prediction accuracy for methane emissions (from 0.085 to 0.301) and methane ratio (from 0.063 to 0.216) compared to the GM model. This improvement was less pronounced but still evident when using PCA components explaining 50% of the variation (333 PCs), with accuracies increasing from 0.085 to 0.230 for methane and from 0.063 to 0.102 for methane ratio compared to the GM model. However, models utilizing higher proportions of variation (75%—640 PCs and 95%—933 PCs) showed substantially lower genomic prediction accuracies than the GM model, with values close to zero for these traits. For LWT, the GM model generally performed better than the NN-GBLUP models, with the exception of the model explaining 95% of microbial variation, which showed slightly higher genomic prediction accuracy. For CO_2_ predictions generally had low accuracies close to zero for both GM and NN-GBLUP models. The dispersion bias for methane emissions was 0.681 for GM, increasing to 1.697 with 25% PCA and improving to 0.904 with 50% PCA. For methane ratio, dispersion bias was 0.472 for GM, improving to 1.160 with 25% PCA and 0.395 with 50% PCA. Scatter plots of predicted versus observed values for methane emissions and methane ratio are shown in Additional file 7, Figure S5.

#### RFI Lucerne group

**Fig. 4:**
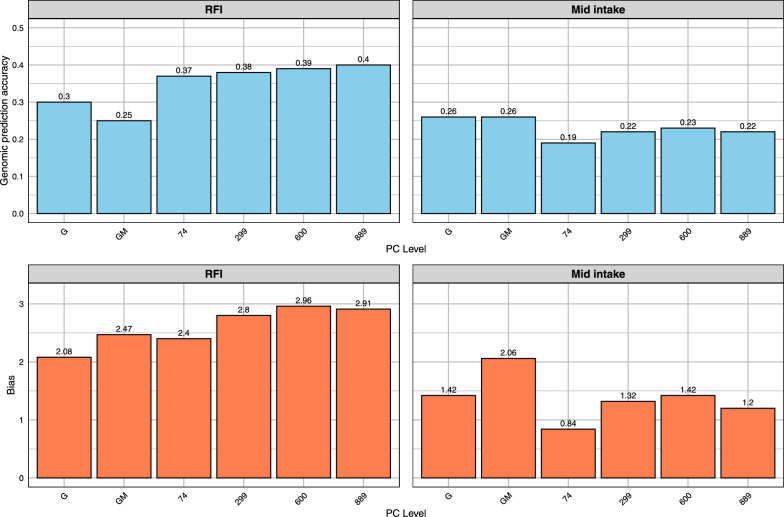
Genomic prediction accuracy and dispersion bias for RFI Lucerne group (2016 Testing). Traits: RFI, mid intake. Methods: G (GBLUP), GM (Method 2), NN-GBLUP (74, 299, 600 PCs)

For residual feed intake (RFI), the baseline GM achieved a genomic prediction accuracy of 0.25 (Fig. 4). The NN-GBLUP model increased genomic prediction accuracy to 0.37 when incorporating PCA components explaining 25% of rumen microbiome variation (74 PCs) and achieved a higher genomic prediction accuracy of 0.40 with PCA components explaining 95% of rumen microbiome variation (889 PCs). However, predictions for average mid-intake did not show improvement, with accuracies ranging from 0.19 to 0.23 across different percentages of explained variation, compared to 0.26 in the GM model. For RFI, the dispersion bias was 2.47 for GM and 2.4 for principal components that explains 25% of variation (25%PCA). For mid-intake, dispersion bias improved from 2.06 (GM) to 0.84 (25% PCA). Scatter plots of predicted versus observed values for RFI are shown in Additional file 7, Figure S5.

### Five-fold cross-validation results

#### Methane Grass group

**Fig. 5:**
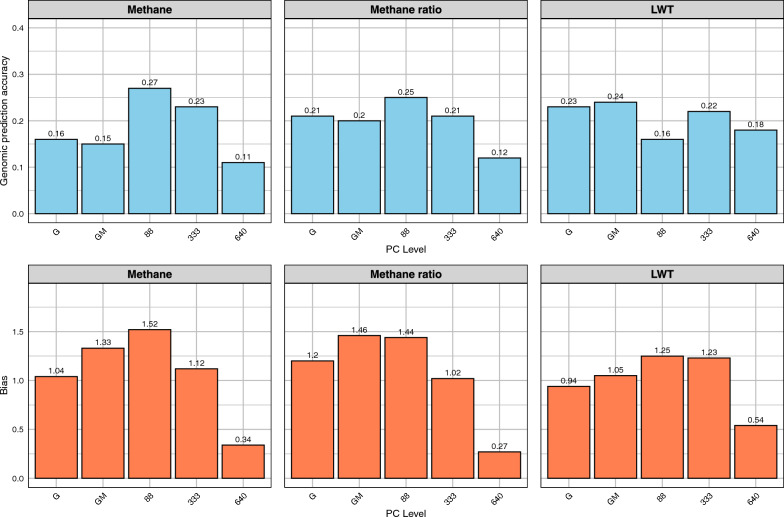
Genomic prediction accuracy and dispersion bias for Methane Grass group (Five-Fold Cross-Validation). Traits: Methane, Methane Ratio, LWT. Methods: G (GBLUP), GM (Method 2), NN-GBLUP (88, 333, 640, 933 PCs)

As shown in Fig. 5, the NN-GBLUP model incorporating PCA components explaining 25% of the rumen microbiome variation (88 PCs) substantially improved the genomic prediction accuracy for methane emissions (from 0.149 to 0.267) compared to the GM model. This improvement was less pronounced but still evident when using PCA components explaining 50% of the variation (333 PCs), with accuracy increasing from 0.149 to 0.229 for methane emissions. For methane ratio, the accuracy increased from 0.202 in the GM model to 0.252 in the NN-GBLUP model explaining 25% of variation, and to 0.212 with 50% of variation explained. However, models utilizing higher proportions of variation (75%—640 PCs and 95%—933 PCs) showed reduced genomic prediction accuracies for these traits. For LWT, unlike in the train-test validation, the GM model (0.243) generally outperformed the NN-GBLUP models. CO_2_ predictions yielded low accuracies for both GM and NN-GBLUP models across all levels of explained variation. For methane emissions, dispersion bias was 1.327 for GM, 1.519 for 25% PCA, and improved to 1.120 with 50% PCA. For methane ratio, both GM and 25% PCA showed similar dispersion bias (1.459 and 1.442), improving to 1.016 with 50% PCA.

#### RFI Lucerne group

**Fig. 6:**
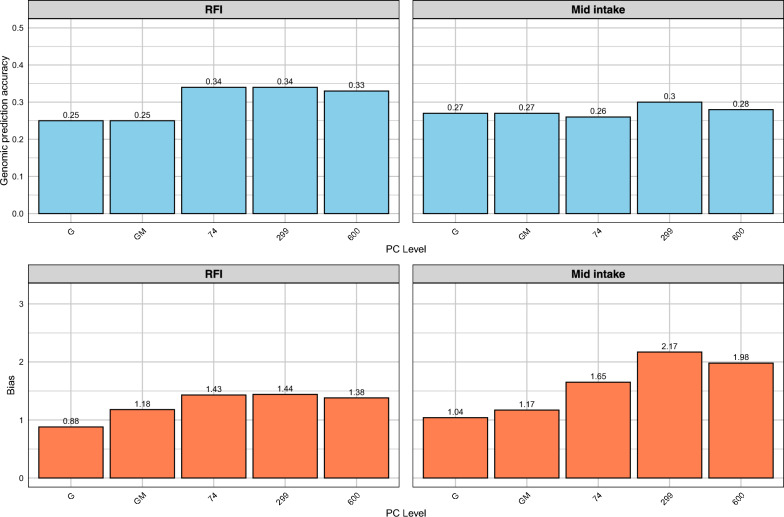
Genomic prediction accuracy and dispersion bias for RFI Lucerne group (Five-Fold Cross-Validation). Traits: RFI, mid intake. Methods: G (GBLUP), GM (Method 2), NN-GBLUP (74, 299, 600 PCs)

For residual feed intake (RFI), the baseline GM model showed a genomic prediction accuracy of 0.25 (Fig. 6). The NN-GBLUP model improved genomic prediction accuracy to 0.34 when incorporating PCA components explaining 25% of rumen microbiome variation (74 PCs). The accuracy remained stable at 0.34 with 50% PCA components (299 PCs) and slightly decreased to 0.33 with 75% PCA components (600 PCs). For mid-intake, the GM model achieved an accuracy of 0.27. The NN-GBLUP model with 25% PCA components yielded a similar accuracy of 0.26, increasing to 0.31 with 50% PCA components and 0.28 with 75% PCA components. For RFI, dispersion bias was 1.18 for GM and increased to 1.43 with 25% PCA and 1.44 with 50% PCA. Similarly, for mid-intake, dispersion bias rose from 1.17 (GM) to 1.65 (25% PCA) and 2.17 (50% PCA). Detailed model performance, dispersion biases, and comparisons across validation strategies can be found in Additional file 1, Table S1 and Additional file 2, Table S2. The standard errors, along with estimates of correlation and bias, are also provided in Additional file 1, Table S1 and Additional file 2, Table S2.

To address methodological considerations regarding compositional data analysis in microbiome studies, we conducted a parallel analysis using the Centered Log-Ratio (CLR) transformation for microbiome preprocessing. Due to the compositional nature of reference-free rumen microbiome data [38], counts were incremented by 1 (pseudo count) to handle zeros, then transformed using the CLR transformation method [39]. The CLR-transformed data were standardized (mean = 0, variance = 1) within each rumen cohort (animals from the same flock, birth year, sex, and sequencing batch). The CLR analysis yielded consistent results with our original transformation method across all aspects of our study. The PCA decomposition showed similar patterns, with comparable percentages of variation explained by the principal components at the 25%, 50%, 75%, and 95% thresholds (see Additional file 8, Figure S6). Microbiability estimates across all traits remained consistent between transformation methods, with differences of less than 0.03 (see Additional file 9, Figure S7). For the GM model under train-test validation and NN-GBLUP for 25% and 50% PCA components, prediction accuracies were similar to those obtained with our original transformation and the dispersion bias was also similar (see Additional file 10, Figure S8 and Additional file 11, Figure S9). Most importantly, the NN-GBLUP models with PCA components explaining 25% and 50% of variation maintained their higher genomic prediction accuracy for methane traits compared to the GM model, with comparable accuracy improvements (see Additional file 12, Figure S10 and Additional file 13, Figure S11). These findings indicate that our conclusions regarding microbiome contribution to trait variation and the relative efficacy of different statistical modelling approaches (GM and NN-GBLUP) are consistent across the two transformation methods. All results including main findings and supplementary materials are deployed in the following Shiny app: https://setalemu.shinyapps.io/genomics_app_deploy/, with source code available at https://github.com/AgResearch/genomics-prediction-app/.

## Discussion

This study aimed to increase the accuracy of breeding values for methane emissions and feed intake by integrating rumen microbiome composition (RMC) data and host genotype data with phenotypic measures. Our research was motivated by the challenges inherent in collecting extensive methane and feed intake phenotypes, particularly in pasture-based sheep and beef meat production systems where individual measurements are often impractical. While one way to increase accuracy is to expand the reference population, this approach is economically costly for traits like methane emissions and residual feed intake (RFI). Instead, we explored the use of RMC information as an intermediate trait to improve genomic predictions via Neural Network Genomic Best Linear Unbiased Prediction (NN-GBLUP). To address computational challenges associated with the high dimensionality of rumen microbiome data, Principal Component Analysis (PCA) was employed to reduce data dimensionality, with principal components demonstrating effective retention of critical information. Subsequently, we integrated PCA-reduced RMC information as intermediate traits into an NN-GBLUP model. This approach improved genomic prediction accuracy for methane emissions and RFI compared to traditional GBLUP models, both with and without rumen microbiome information as a non-genetic random term. Optimal performance was achieved with PCA components explaining 25% of the rumen microbiome variation. These findings highlight the potential of integrating RMC information into genomic prediction models to improve the accuracy and efficiency of livestock breeding programs, supporting sustainability and productivity goals.

### Effectiveness of PCA in capturing RMC information

PCA demonstrated high efficiency in extracting essential signals from the comprehensive microbiome dataset. To our knowledge, this is the first microbiome study to demonstrate that microbiability estimates derived from PCA components explained 100% of the variation, matching results from the full dataset. This finding agrees with earlier research [40], that demonstrated capacity of PCA to reduce SNP data dimensionality while maintaining genomic breeding value accuracy. Our results indicate that PCA may also improve the detection of underlying microbiome signals by effectively removing irrelevant information. The consistency of our findings across distinct microbial tag datasets for methane emissions and residual feed intake (RFI) suggests the potential for broad application of PCA to various microbial datasets in ruminant studies. This observation aligns with previous work [40] that noted similar PCA effectiveness across different cattle breeds, further emphasizing its robustness. However, further validation across a wider range of datasets would be beneficial in establishing the full broad applicability of this approach. Moreover, PCA offered significant computational advantages, substantially reducing processing time compared to traditional methods that directly analyze all marker genotypes, as reported in previous studies [40]. Importantly, the dimensionality reduction achieved through PCA enabled the integration of over 200,000 rumen microbial tags as intermediate features in our Bayesian neural network model, which would have been computationally prohibitive using conventional approaches.

### Genomic prediction accuracy using rumen microbiome PCA

Building on the successful application of PCA for microbiability estimation, we investigated its impact on genomic prediction accuracy. Our results revealed that incorporating PCA-reduced RMC information as observed intermediate traits within a Neural Network GBLUP (NN-GBLUP) model significantly increased genomic prediction accuracy for methane emissions and RFI compared to baseline GBLUP models. This improvement in accuracy can be attributed to the NN-GBLUP's ability to jointly utilize both genotype and omics features in a one-step approach, but only when these omics features are biologically relevant to the trait. In contrast, the conventional GBLUP model only utilizes genotype information, missing the effects of the microbiome, while the GM model, despite having access to both omics and genotype data, assumes zero covariance between these features, limiting its ability to capture their joint effects. Even with this joint modeling approach, the NN-GBLUP did not increase prediction accuracy for traits such as CO2 emissions, mid-intake, and live weight, where the rumen microbiome features likely have less biological influence. This differential impact clearly demonstrates that the effectiveness of incorporating intermediate traits in genomic prediction is highly dependent on the specific trait being predicted. The traits that showed improvement (methane emissions and RFI) are known to be strongly influenced by rumen microbial activity, while the others may have less direct microbial involvement or more complex relationships with the microbiome. Furthermore, the improvement in accuracy also suggests that a substantial portion of the microbiome effect on these traits might be controlled by host genetics, a phenomenon previously observed for RFI [22, 24, 41, 42], and for methane [9, 25, 41, 43]. More specifically, Li et al. [22] reported that the relative abundance of about 34% of rumen microbial taxa (59 out of 174) had a heritability estimate (h2) ≥ 0.15, indicating that these microbial features are heritable elements affected by host additive genetics associated with RFI and Roehe et al. [41] further supported this by demonstrating that the archaea: bacteria ratio in the rumen was influenced by host genetics and associated with methane emissions. These studies provide evidence for the genetic basis of the host's influence on rumen microbial composition, which aligns with our findings on improved prediction accuracy when incorporating microbiome information.

The high genomic prediction accuracy using an intermediate trait is supported by recent studies. For instance, Yang et al. [44] reported that a microbiome-enabled genomic selection model significantly outperformed conventional genomic selection for nearly all time-series traits related to plant growth and nitrogen responses. Similarly, Guo et al. [45] demonstrated improved genomic prediction accuracy for malting quality traits in barley when metabolomic information was included as an intermediate trait. These findings, along with our results, suggest a broader applicability of integrating microbiome or other 'omic' data as intermediate traits in genomic prediction models, particularly for traits with a strong microbial component. However, the differential impact on various traits emphasizes the need for a trait-specific approach when incorporating such data. Future research should focus on better understanding the genetic architecture underlying host control of microbiome composition and its subsequent effect on traits of interest.

### Tailoring microbiome integration for trait-specific benefits

The integration of microbial data through PCA components in a Bayesian neural network model demonstrates distinct trends in genomic prediction accuracy for methane emissions and residual feed intake (RFI). For methane traits, optimal prediction accuracy was observed when using PCA components that explain 25% and 50% of the variation (88 and 333 PCA components, respectively). Beyond this threshold, particularly with PCA components explaining 75% and 95% of the variation, additional data did not capture trait-relevant signals, suggesting a maximal threshold of microbial information that is useful for prediction of methane traits. On the other hand, for RFI, prediction accuracy using the Bayesian neural network model showed consistent improvement with the inclusion of more PCA components, yet the gains became marginal and plateaued after explaining 25% of the variation. This suggests that the additional or later principal components might not capture relevant signals of the trait.

To validate that later PCs contribute minimally to trait prediction, we conducted additional analyses using specific ranges of PCA components. For methane traits, components spanning PC 334–640 (50–75% variation) and PC 334–933 (50–95% variation) yielded negligible accuracies (−0.04 and 0.01 respectively). Similarly, for RFI, components from PC 300–600 (50–75% variation) and PC 300–889 (50–95% variation) showed minimal predictive value (accuracies of 0.016 and 0.01). These results confirm that later PCs contribute negligibly to prediction accuracy, and their inclusion can even be detrimental for methane traits.

The contrasting responses to using more PCs between methane and RFI traits may be related to their genetic architectures. To understand these genetic architecture differences, we estimated heritability and microbiability from our data. We found methane traits had lower heritability (h^2^ = 0.18) and moderate microbiability (0.47), making them more susceptible to overfitting when non-informative PCA components are included. In contrast, our estimates for RFI showed higher heritability (h^2^ = 0.33) and stronger microbiability (0.7), providing greater resilience against overfitting, even when weaker or non-informative signals are included. For low-heritability traits like methane, increasing training population size is more effective than increasing model complexity. This is because genomic prediction accuracy depends on heritability, training size, and model complexity—[17, 46]. To demonstrate this in our study, we conducted additional analyses using 640 PCs (75% of variation) but with different training set sizes. Increasing the training population from 791 to 951 individuals (with corresponding test sizes of 261 and 100) improved methane prediction accuracy from 0.05 to 0.12. These findings align with previous studies that for low-heritability traits, the optimal strategy combines adequate training population size with appropriate model complexity [46, 47]. In our specific case, this translates to using PCA components explaining 25–50% of the variation, rather than incorporating additional components, as further increasing model complexity (by including more PCs) does not improve predictive accuracy.

### Impact of validation methods on dispersion bias and accuracy

A comparison between train-test split and cohort-specific five-fold cross-validation reveals important insights into model performance. Five-fold cross-validation demonstrated superior consistency and reduced dispersion bias across all scenarios, including GBLUP without PCA and models incorporating microbiome information at various PCA component levels (25%, 50%). This improvement in dispersion bias reduction is particularly evident when compared to the train-test split approach. The train-test split method, which includes entire cohorts in either the training or testing set, can lead to significant environmental differences between these sets due to year and cohort effects. These systematic variations arise from year-to-year differences in climate conditions, feed quality, and management practices, as well as cohort-specific effects. The concentration of these environmental effects in distinct training and testing sets tends to produce more biased estimates of genetic parameters compared to five-fold cross-validation, where the mixing of cohorts across folds helps average out these environmental effects. In contrast, five-fold cross-validation addresses this issue by dividing each cohort into smaller subsets and ensuring a mix of individuals from different cohorts in each fold. This strategy reduces environmental variance and cohort-specific dispersion bias, as individuals from each cohort are represented across different folds.

The impact of the cross-validation strategy on dispersion bias reduction is particularly evident for traits with significant environmental covariation, such as residual feed intake (RFI) [48, 49]. The dispersion bias for RFI decreased from 2.5 using a train-test split with 25% PCA components to 1.4 with cohort-specific five-fold cross-validation. This reduction brings the estimates closer to having no dispersion bias, highlighting the effectiveness of the five-fold cross-validation approach in mitigating environmental effects across different model types and PCA component levels. These findings underscore the importance of choosing appropriate cross-validation strategies in genomic prediction studies, especially when dealing with traits highly influenced by environmental factors. While the results align with Schrauf et al.’s [50] recommendation of using k-fold cross-validation (k = 5 or 10) for estimating the average predictive performance of genomic models across different subsets of the data, our study specifically highlights its potential in reducing dispersion bias related to environmental effects. The cohort-specific five-fold cross-validation approach used in this study showed improved performance in handling environmental variations compared to the train-test split method.

### Future studies

While our approach shows promise, it is important to note the limitations of our study. The relatively small sample size (1051 animals for methane traits and 984 for RFI) likely influences our results, particularly the observation that genomic prediction accuracy does not increase with larger numbers of principal components. This limitation may explain why we see optimal performance with PCA components explaining only 25% or 50% of the rumen microbiome variation. To address this, larger-scale studies are needed to validate our findings and determine if more microbiome information could be beneficial with increased sample sizes.

In an ideal scenario, model development would involve three distinct partitions: (1) a training set for parameter estimation, (2) a validation set for hyperparameter selection, and (3) an independent test set for final evaluation. In applied settings with juvenile prediction, the appropriate dimension reduction would be determined through cross-validation within the reference population. However, due to our limited sample size, we employed a two-partition approach (training and test sets) and fitted NN-GBLUP models with PCA components explaining pre-specified percentages of RMC variation. We acknowledge that selecting optimal PCA components based on test set performance could confer an unfair advantage over the applied GBLUP model, an advantage that would not be available in practical implementation with juvenile prediction. Nevertheless, our comparison between GM and NN-GBLUP models provides indirect evidence of the neural network's predictive power, as both models incorporate identical genomic and microbiome information sources. The NN-GBLUP model did not have any extra information advantage over the GM model, yet consistently outperformed it, particularly with components explaining 25% of the variation for methane emissions and residual feed intake. This demonstrates the neural network's superior ability to capture complex relationships between host genetics and microbiome data, even with substantial dimension reduction. We recommend that future studies with larger datasets employ appropriate three-way data partitioning when evaluating dimension reduction techniques.

While our reference-free approach to quantifying rumen microbial tags effectively estimates the combined impact of the rumen microbiome, it lacks specificity in identifying the most influential microbial taxa for traits of interest. It should be noted that we did not directly compare the reference-free approach with reference-based methods in this study. Although reference-based approaches offer advantages in taxonomic assignment and functional interpretation, the reference-free approach captures the full genetic diversity present in the samples, including potentially novel or uncultured microbes that are particularly common in ruminant microbiomes. Nevertheless, a direct comparative analysis between these approaches would be valuable for determining the optimal methodology and represents an important direction for future research.

Furthermore, our current study focused on linear associations through PCA-based dimensionality reduction, which was appropriate given our sample size constraints. However, this presents an opportunity to explore non-linear interactions within the rumen microbiome in future research with larger datasets. To pursue this research direction, two approaches warrant further investigation. The first approach involves adopting reference-based methodologies, which would inherently generate lower-dimensional data through taxonomic classification. This natural dimensionality reduction would facilitate direct modelling of non-linear interactions without necessitating additional computational reduction steps. Reference-based methods offer enhanced biological interpretability and taxonomic resolution with computationally manageable dimensions. Alternatively, as a second approach, maintaining the reference-free methodology, which comprehensively captures microbial genetic diversity, while implementing non-linear dimensionality reduction techniques such as kernel-based PCA [51] or autoencoders [52]. These algorithms can identify non-linear relationships that linear PCA methods may not address. While reference-free methods with non-linear dimensionality reduction preserve comprehensive microbial diversity and capture complex interactions, they require additional computational steps for dimensionality reduction. Irrespective of the methodological approach selected, increased sample sizes remain imperative for robust statistical power in modelling complex, non-linear relationships between rumen microbial populations and phenotypic traits of interest.

## Conclusion

This study demonstrates that Principal Component Analysis (PCA) can effectively reduce high-dimensional rumen microbiome data while preserving essential biological information. The integration of these reduced-dimensional features as intermediate traits within a Bayesian neural network model substantially improved genomic prediction accuracy for methane emissions and feed efficiency traits in sheep. Importantly, while using principal components explaining 25–50% of variation improves genomic prediction accuracy, higher percentages (75–95% of variation) notably diminish prediction accuracies, especially for methane traits. Optimal selection of principal components is crucial, as it increases genomic prediction accuracy, as demonstrated by improved results when using principal components explaining lower percentages of variation. This approach provides an effective strategy for optimizing livestock breeding programs to improve efficiency and reduce environmental impact.

## Supplementary Information

Additional file 1. Genomic prediction accuracy and bias for methane (grass diet) and residual feed intake (lucerne diet) traits using a training population born in 2014 and 2015, and a test population born in 2016.

Additional file 2. Genomic prediction accuracy and bias for methane (grass diet) and residual feed intake (lucerne diet) traits using five-fold cohort-based cross-validation.

Additional file 3. Genomic prediction accuracy for methane (grass diet) using Model 2 (GM) with varying proportions of PCA-reduced microbiome data (train-test validation).

Additional file 4. Genomic prediction accuracy for residual feed intake (lucerne diet) using Model 2 (GM) with varying proportions of PCA-reduced microbiome data (train-test validation).

Additional file 5. Genomic prediction accuracy for methane (grass diet) using Model 2 (GM) with varying proportions of PCA-reduced microbiome data (five-fold cross-validation).

Additional file 6. Genomic prediction accuracy for residual feed intake (lucerne diet) using Model 2 (GM) with varying proportions of PCA-reduced microbiome data (five-fold cross-validation).

Additional file 7. Scatter plot of predicted genomic breeding values using 88 principal components (25% of microbial variation) for methane, methane ratio, and 74 principal components (25% of microbial variation) for train-test cross-validation.

Additional file 8. Comparison of Cumulative Variance Explained by Principal Components Using Log and CLR Transformations of Rumen Microbial Profiles.

Additional file 9. Comparison of Microbiability Estimates Using Log and CLR Transformations of Rumen Microbial Profiles across Different Variance Thresholds.

Additional file 10. Genomic Prediction Accuracy and Bias for Methane Group Traits Using CLR and Log Transformations with the GM Model.

Additional file 11. Genomic Prediction Accuracy and Bias for RFI Group Traits Using CLR and Log Transformations with the GM Model.

Additional file 12. Comparison of NN-GBLUP Genomic Prediction Accuracy Using CLR versus Log Transformation across All Traits.

Additional file 13. Comparison of NN-GBLUP Dispersion Bias Using CLR versus Log Transformation across All Traits.

## Data Availability

The data used in this study were from a previous published paper by Hess et al. [13]. The original sequence data is available in the NCBI Short Read Archive (SRA) database under BioProject ID PRJNA859547. The phenotypic and genotypic data analysed during the current study are available from Sheep Improvement Limited or AgResearch, but restrictions apply to the availability of these data, which were used under license for the current study, and so are not publicly available.
