# Supplementary material for: Improving genomic prediction accuracy for methane emission and feed efficiency in sheep: integrating rumen microbial PCA with host genomic variation using neural network GBLUP (NN-GBLUP)
Source: Genet Sel Evol. 2025 Jul 17;57:41. doi: 10.1186/s12711-025-00987-x (PMC12273308; doi:10.1186/s12711-025-00987-x)
Supplement: Supplementary file 1 — Additional file 1. Genomic prediction accuracy and bias for methane (grass diet) and residual feed intake (lucerne diet) traits using a training population born in 2014 and 2015, and a test population born in 2016. [file 12711_2025_987_MOESM1_ESM.docx]

Table S1 Genomic prediction accuracy and dispersion bias for three models for two group of traits (Methane Grass group and Residual feed intake (RFI) Lucerne Group) using a training population born I year 2014 and 2015 and Test population year 2016

| Trait Group | Model^1^ | Trait | ^2^$\boldsymbol{r}_{\boldsymbol{Ap}}$ | Dispersion bias |
| --- | --- | --- | --- | --- |
| Methane  Grass  Group | G | Methane | 0.114 ± 0.052 | 0.776 ± 0.358 |
|  | GM | Methane | 0.085 ± 0.052 | 0.681 ± 0.420 |
|  | PC88 | Methane | 0.301 ± 0.048 | 1.697 ± 0.284 |
|  | PC333 | Methane | 0.230 ± 0.050 | 0.904 ± 0.202 |
|  | PC640 | Methane | 0.051 ± 0.052 | 0.092 ± 0.095 |
|  | PC933 | Methane | 0.056 ± 0.052 | 0.081 ± 0.076 |
|  | G | Methane ratio | 0.083 ± 0.052 | 0.551 ± 0.349 |
|  | GM | Methane ratio | 0.063 ± 0.052 | 0.472 ± 0.396 |
|  | PC88 | Methane ratio | 0.216 ± 0.050 | 1.160 ± 0.277 |
|  | PC333 | Methane ratio | 0.102 ± 0.052 | 0.395 ± 0.204 |
|  | PC640 | Methane ratio | 0.025 ± 0.053 | 0.007 ± 0.014 |
|  | PC933 | Methane ratio | 0.015 ± 0.053 | 0.000 ± 0.001 |
|  | G | LWT | 0.197 ± 0.051 | 1.307 ± 0.343 |
|  | GM | LWT | 0.192 ± 0.051 | 1.242 ± 0.336 |
|  | PC88 | LWT | 0.214 ± 0.050 | 1.304 ± 0.314 |
|  | PC333 | LWT | 0.122 ± 0.052 | 0.485 ± 0.208 |
|  | PC640 | LWT | 0.123 ± 0.052 | 0.309 ± 0.131 |
|  | PC933 | LWT | 0.213 ± 0.050 | 0.637 ± 0.154 |
|  | G | CO2 | 0.065 ± 0.052 | 0.359 ± 0.291 |
|  | GM | CO2 | 0.071 ± 0.052 | 0.429 ± 0.317 |
|  | PC88 | CO2 | 0.084 ± 0.052 | 1.384 ± 0.866 |
|  | PC333 | CO2 | 0.022 ± 0.053 | 0.363 ± 0.859 |
|  | PC640 | CO2 | 0.045 ± 0.053 | 0.800 ± 0.930 |
|  | PC933 | CO2 | 0.07 ± 0.053 | 1.180 ± 1.627 |
| RFI  Lucerne  Group | G | RFI | 0.298 ± 0.046 | 2.079 ± 0.335 |
|  | GM | RFI | 0.247 ± 0.047 | 2.470 ± 0.489 |
|  | PC74 | RFI | 0.374 ± 0.043 | 2.404 ± 0.301 |
|  | PC299 | RFI | 0.380 ± 0.043 | 2.805 ± 0.344 |
|  | PC600 | RFI | 0.387 ± 0.043 | 2.964 ± 0.355 |
|  | PC889 | RFI | 0.396 ± 0.042 | 2.909 ± 0.340 |
|  | G | Mid intake | 0.262 ± 0.047 | 1.424 ± 0.264 |
|  | GM | Mid intake | 0.256 ± 0.047 | 2.056 ± 0.391 |
|  | PC74 | Mid intake | 0.188 ± 0.048 | 0.838 ± 0.221 |
|  | PC299 | Mid intake | 0.218 ± 0.048 | 1.315 ± 0.296 |
|  | PC600 | Mid intake | 0.227 ± 0.048 | 1.421 ± 0.306 |
|  | PC889 | Mid intake | 0.216 ± 0.048 | 1.201 ± 0.273 |

^1^Model Descriptions:

G: Genomics model (using only genomic information) GM: Genomics + Microbiome model (combining genomic and full microbiome data) PC*: Bayesian neural network models incorporating varying degrees of microbial variation as intermediate trait

For Methane Grass Group:

- PC88: 25% of microbial variation
- PC333: 50% of microbial variation
- PC640: 75% of microbial variation
- PC933: 95% of microbial variation

For RFI Lucerne Group:

- PC74: 25% of microbial variation
- PC299: 50% of microbial variation
- PC600: 75% of microbial variation
- PC889: 95% of microbial variation

^2^$r_{Ap}$ Genomic prediction accuracy: Correlation between estimate breeding value with adjusted phenotype
