# Supplementary material for: Improving genomic prediction accuracy for methane emission and feed efficiency in sheep: integrating rumen microbial PCA with host genomic variation using neural network GBLUP (NN-GBLUP)
Source: Genet Sel Evol. 2025 Jul 17;57:41. doi: 10.1186/s12711-025-00987-x (PMC12273308; doi:10.1186/s12711-025-00987-x)
Supplement: Supplementary file 2 — Additional file 2. Genomic prediction accuracy and bias for methane (grass diet) and residual feed intake (lucerne diet) traits using five-fold cohort-based cross-validation. [file 12711_2025_987_MOESM2_ESM.docx]

Table S2 Genomic prediction accuracy and Dispersion bias for three models for two group of traits (Methane Grass group and Residual feed intake (RFI) Lucerne Group) using a fivefold cohort based cross validation

| Trait Group | ^1^Model | Trait | ^2^$\boldsymbol{r}_{\boldsymbol{Ap}}$ | Dispersion bias |
| --- | --- | --- | --- | --- |
|  |  |  |  |  |
| Methane  Grass  Group | G | Methane | 0.159 ± 0.081 | 1.035 ± 0.631 |
|  | GM | Methane | 0.149 ± 0.073 | 1.327 ± 0.771 |
|  | PC88 | Methane | 0.267 ± 0.070 | 1.519 ± 0.407 |
|  | PC333 | Methane | 0.229 ± 0.087 | 1.120 ± 0.440 |
|  | PC640 | Methane | 0.110 ± 0.069 | 0.337 ± 0.218 |
|  | G | Methane ratio | 0.210 ± 0.052 | 1.197 ± 0.459 |
|  | GM | Methane ratio | 0.202 ± 0.054 | 1.459 ± 0.542 |
|  | PC88 | Methane ratio | 0.252 ± 0.054 | 1.442 ± 0.339 |
|  | PC333 | Methane ratio | 0.212 ± 0.070 | 1.016 ± 0.296 |
|  | PC640 | Methane ratio | 0.118 ± 0.036 | 0.275 ± 0.091 |
|  | G | LWT | 0.232 ± 0.054 | 0.945 ± 0.242 |
|  | GM | LWT | 0.243 ± 0.064 | 1.049 ± 0.301 |
|  | PC88 | LWT | 0.162 ± 0.088 | 1.250 ± 0.816 |
|  | PC333 | LWT | 0.220 ± 0.094 | 1.227 ± 0.578 |
|  | PC640 | LWT | 0.178 ± 0.090 | 0.541 ± 0.286 |
|  | G | CO2 | 0.123 ± 0.028 | 0.716 ± 0.178 |
|  | GM | CO2 | 0.135 ± 0.030 | 0.899 ± 0.260 |
|  | PC88 | CO2 | -0.033 ± 0.058 | -0.502 ± 1.013 |
|  | PC333 | CO2 | -0.016 ± 0.059 | -0.222 ± 1.040 |
|  | PC640 | CO2 | -0.003 ± 0.068 | 0.011 ± 1.159 |
| RFI  Lucerne  Group | G | RFI | 0.255 ± 0.049 | 0.879 ± 0.231 |
|  | GM | RFI | 0.248 ± 0.040 | 1.182 ± 0.156 |
|  | PC74 | RFI | 0.336 ± 0.086 | 1.435 ± 0.432 |
|  | PC299 | RFI | 0.343 ± 0.078 | 1.444 ± 0.427 |
|  | PC600 | RFI | 0.330 ± 0.079 | 1.377 ± 0.411 |
|  | G | Mid intake | 0.273 ± 0.036 | 1.036 ± 0.144 |
|  | GM | Mid intake | 0.275 ± 0.060 | 1.169 ± 0.314 |
|  | PC74 | Mid intake | 0.258 ± 0.043 | 1.647 ± 0.342 |
|  | PC299 | Mid intake | 0.305 ± 0.063 | 2.167 ± 0.682 |
|  | PC600 | Mid intake | 0.280 ± 0.031 | 1.980 ± 0.315 |

Model Descriptions:

G: Genomics model (using only genomic information) GM: Genomics + Microbiome model (combining genomic and full microbiome data) PC*: Bayesian neural network models incorporating varying degrees of microbial variation

For Methane Grass Group:

- PC88: 25% of microbial variation
- PC333: 50% of microbial variation
- PC640: 75% of microbial variation

For RFI Lucerne Group:

- PC74: 25% of microbial variation
- PC299: 50% of microbial variation
- PC600: 75% of microbial variation
- ^2^$r_{Ap}$ Genomic prediction accuracy: Correlation between estimate breeding value with adjusted phenotype
