# Supplementary figures and images for: Improving genomic prediction accuracy for methane emission and feed efficiency in sheep: integrating rumen microbial PCA with host genomic variation using neural network GBLUP (NN-GBLUP)
Source: Genet Sel Evol. 2025 Jul 17;57:41. doi: 10.1186/s12711-025-00987-x (PMC12273308; doi:10.1186/s12711-025-00987-x)

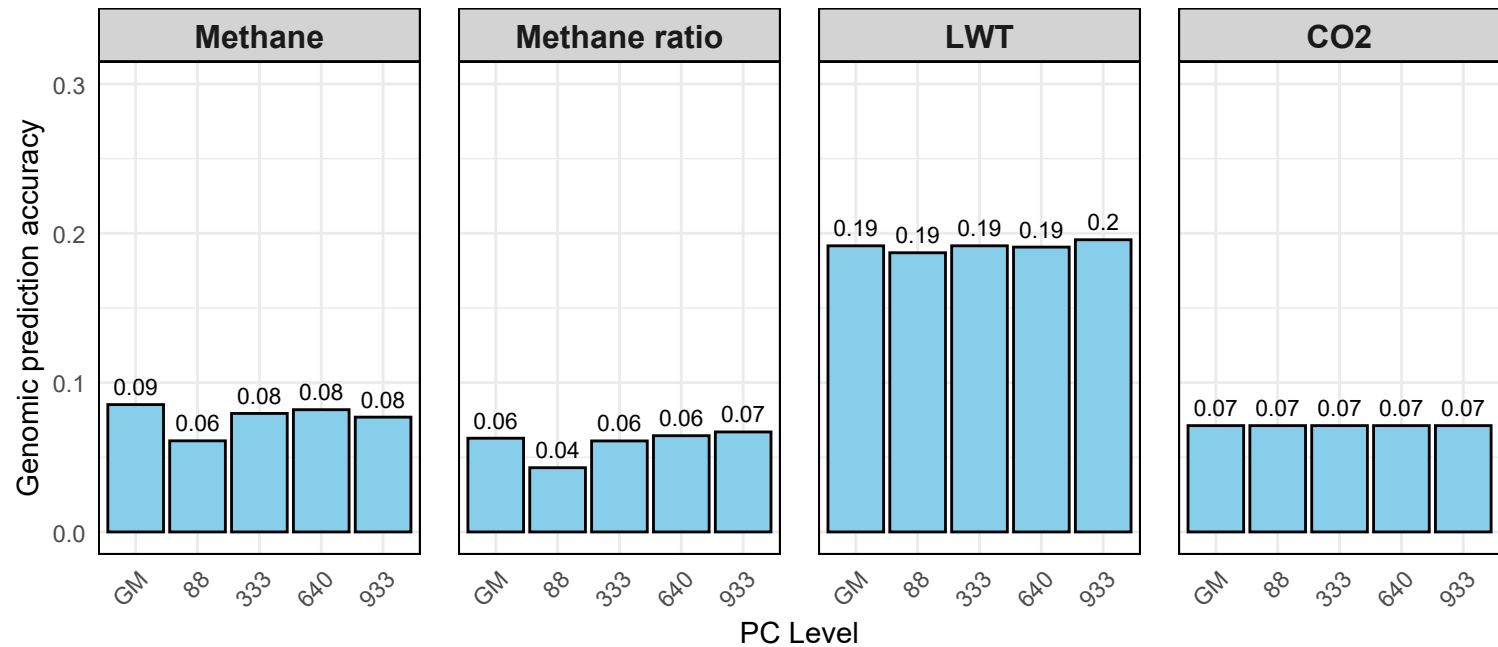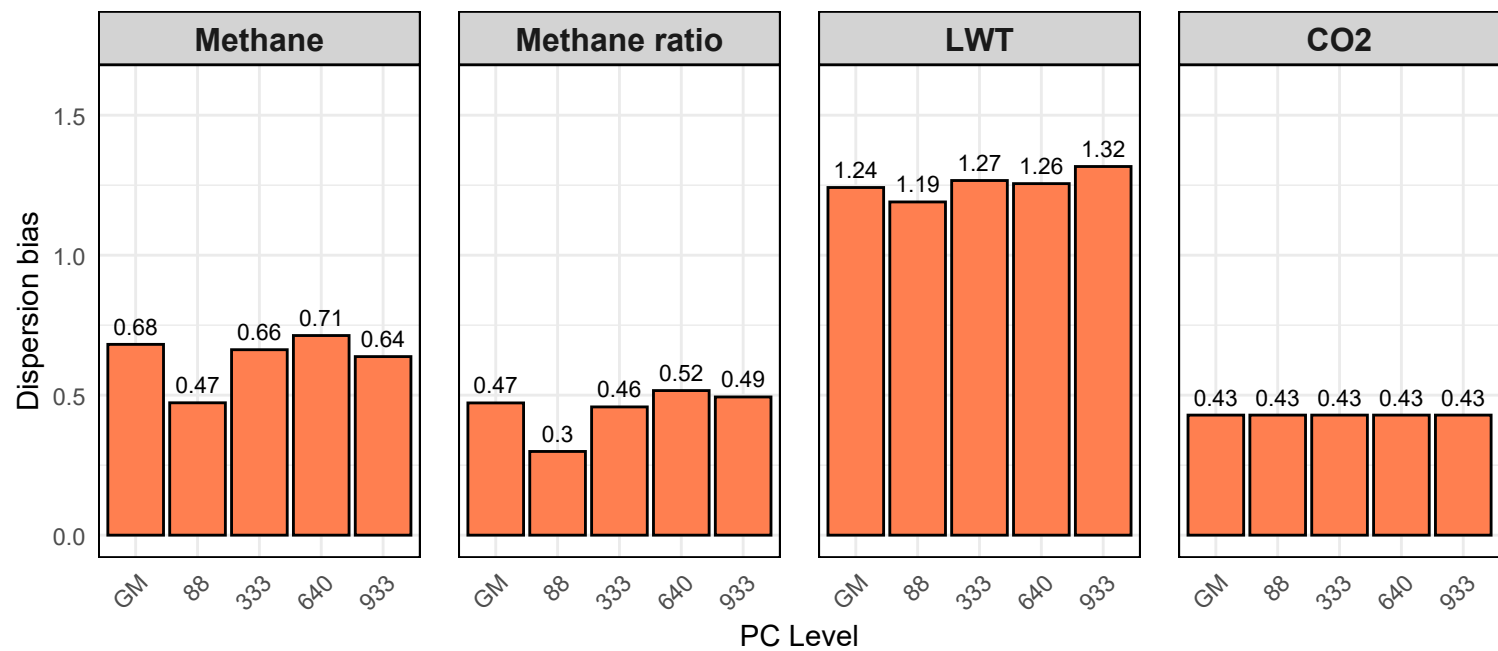

Supplement: Supplementary file 3 — Additional file 3. Genomic prediction accuracy for methane (grass diet) using Model 2 (GM) with varying proportions of PCA-reduced microbiome data (train-test validation). [file 12711_2025_987_MOESM3_ESM.pdf]

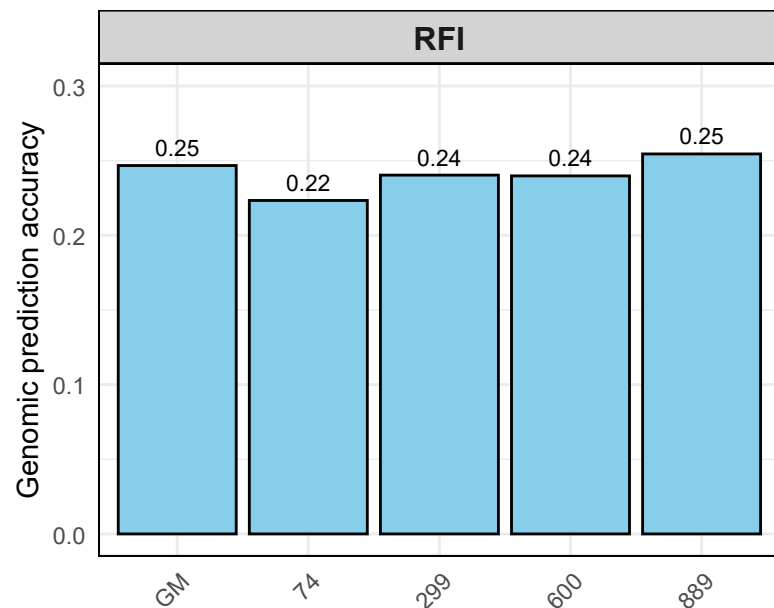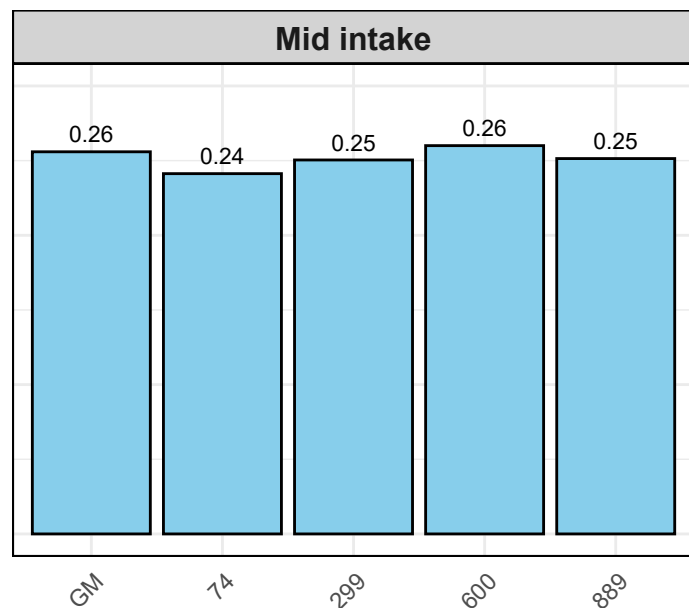

PC Level

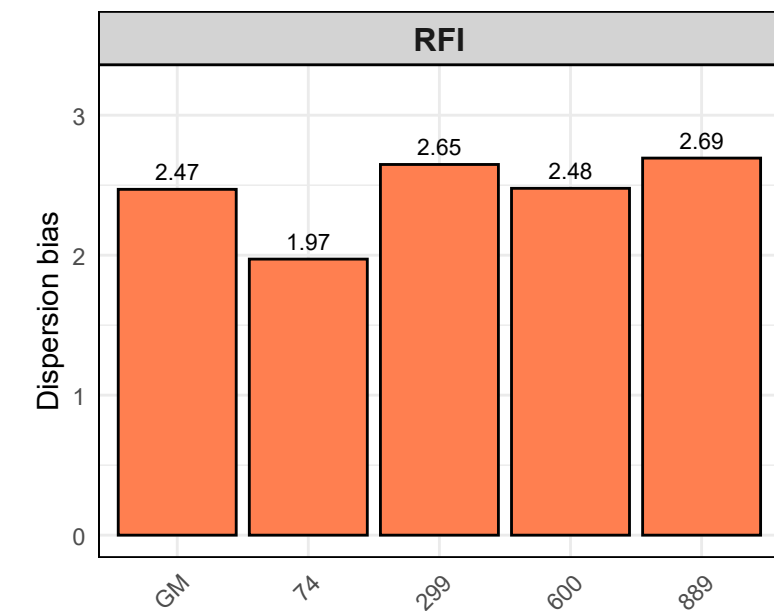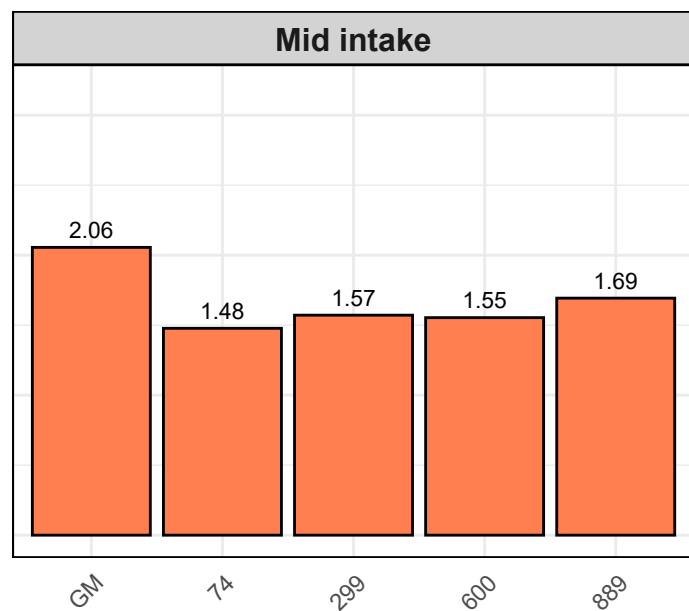

PC Level

Supplement: Supplementary file 4 — Additional file 4. Genomic prediction accuracy for residual feed intake (lucerne diet) using Model 2 (GM) with varying proportions of PCA-reduced microbiome data (train-test validation). [file 12711_2025_987_MOESM4_ESM.pdf]

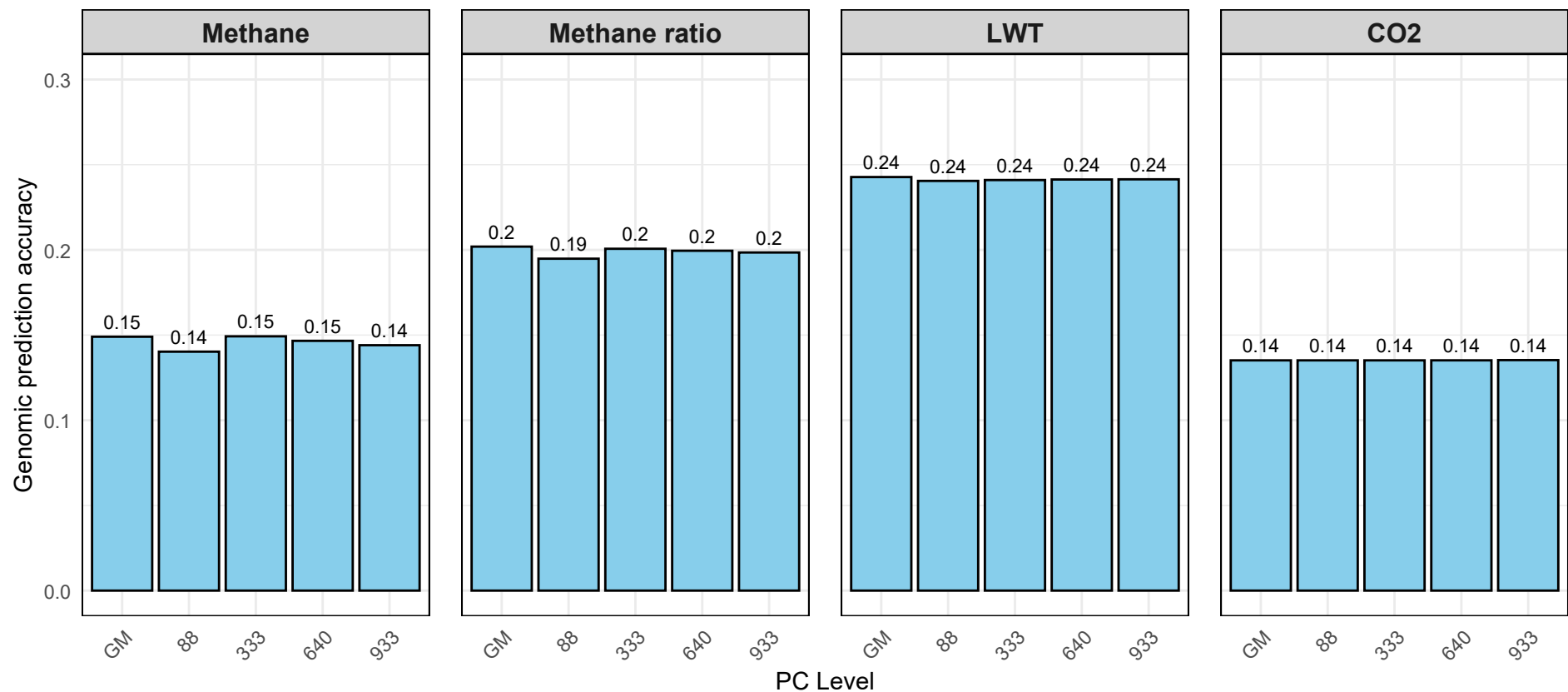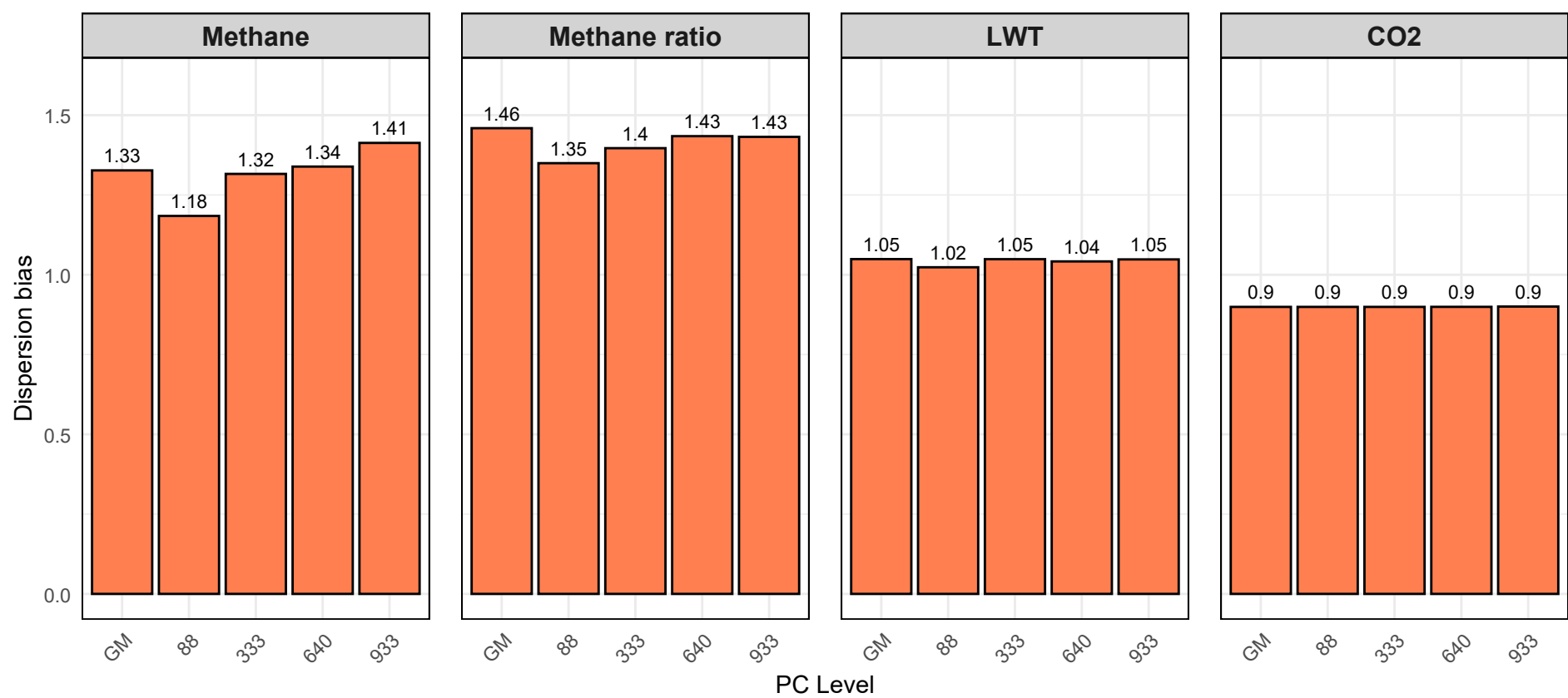

Supplement: Supplementary file 5 — Additional file 5. Genomic prediction accuracy for methane (grass diet) using Model 2 (GM) with varying proportions of PCA-reduced microbiome data (five-fold cross-validation). [file 12711_2025_987_MOESM5_ESM.pdf]

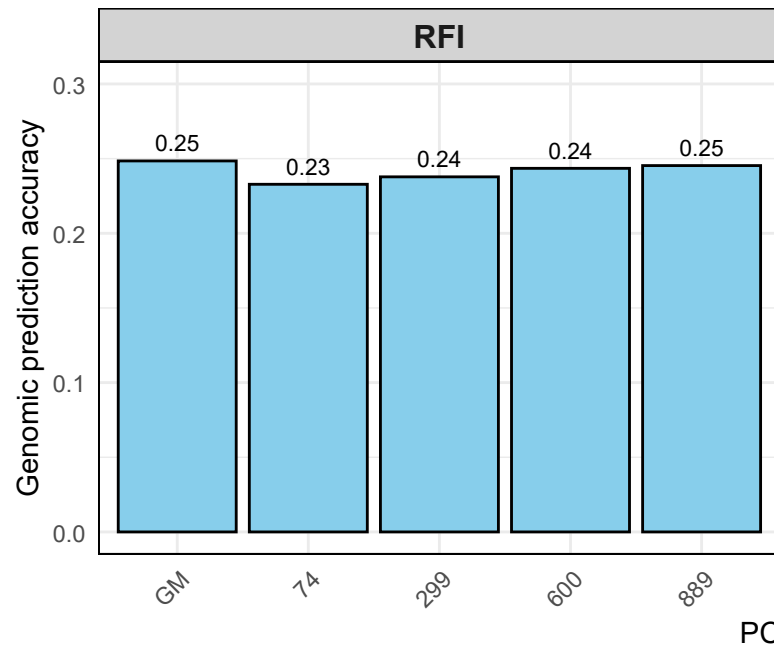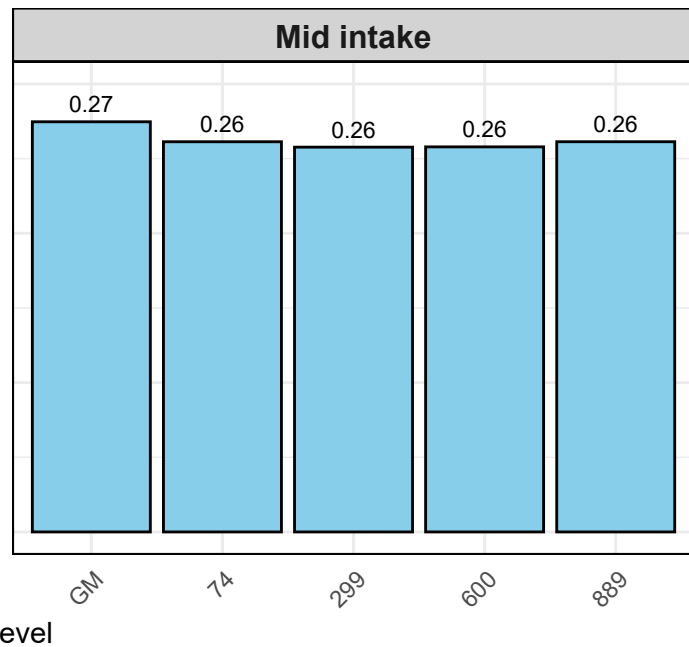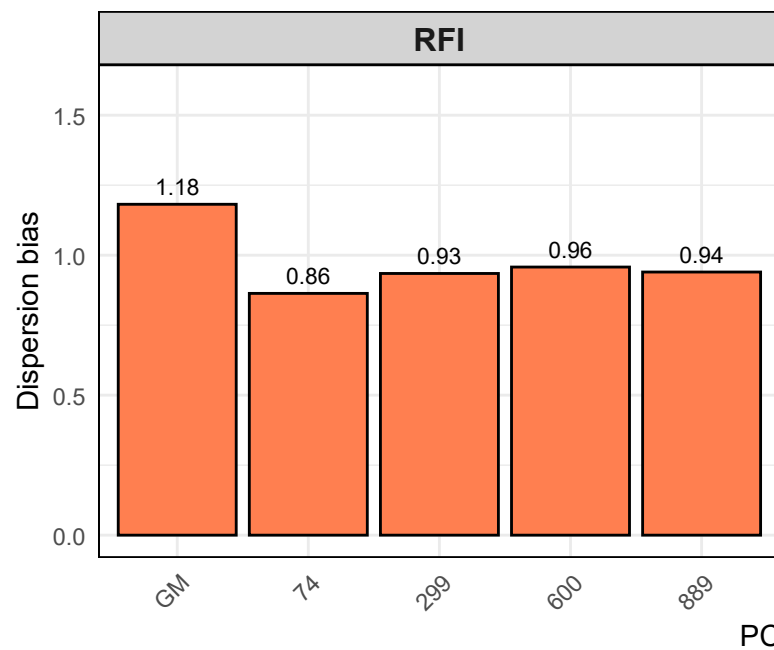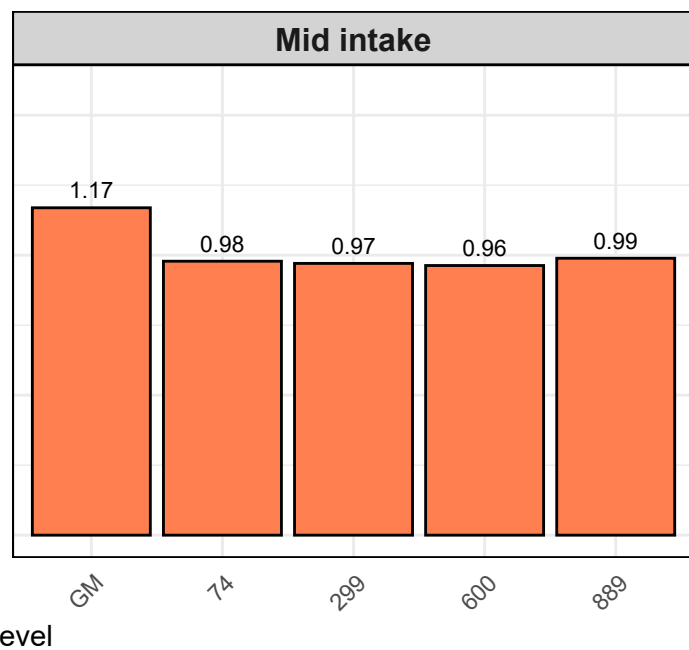

Supplement: Supplementary file 6 — Additional file 6. Genomic prediction accuracy for residual feed intake (lucerne diet) using Model 2 (GM) with varying proportions of PCA-reduced microbiome data (five-fold cross-validation). [file 12711_2025_987_MOESM6_ESM.pdf]

## Methane

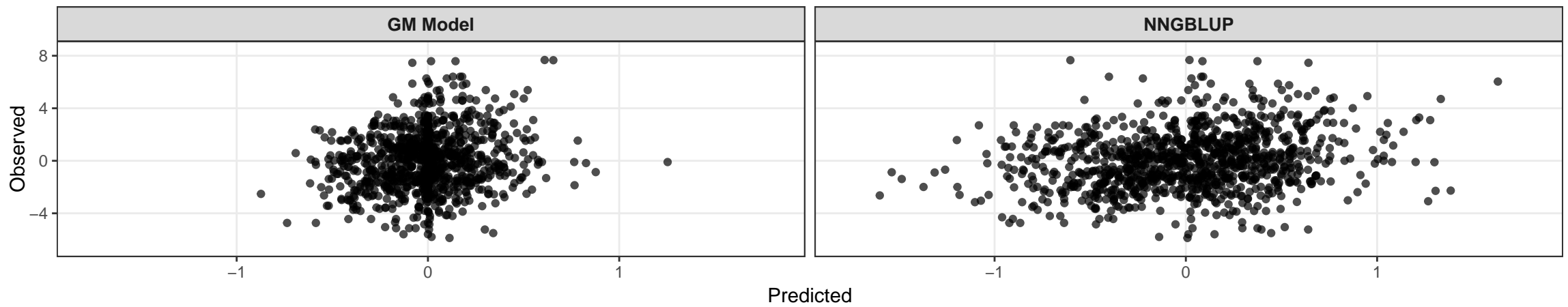

## Methane ratio

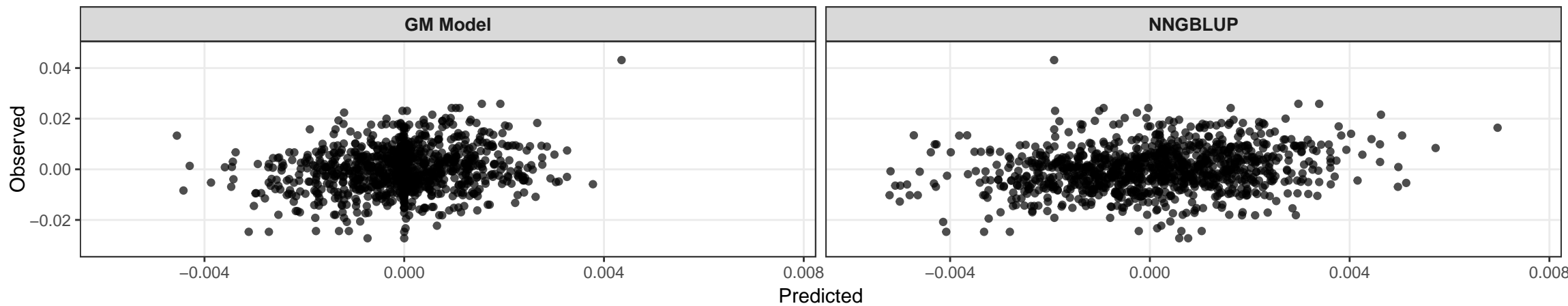

## RFI

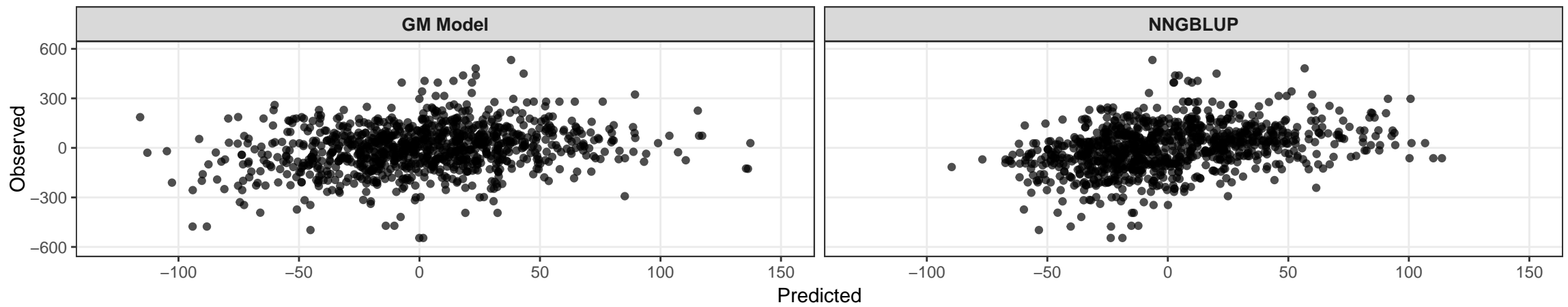

Supplement: Supplementary file 7 — Additional file 7. Scatter plot of predicted genomic breeding values using 88 principal components (25% of microbial variation) for methane, methane ratio, and 74 principal components (25% of microbial variation) for train-test cross-validation. [file 12711_2025_987_MOESM7_ESM.pdf]

# Variance Comparison: All Traits

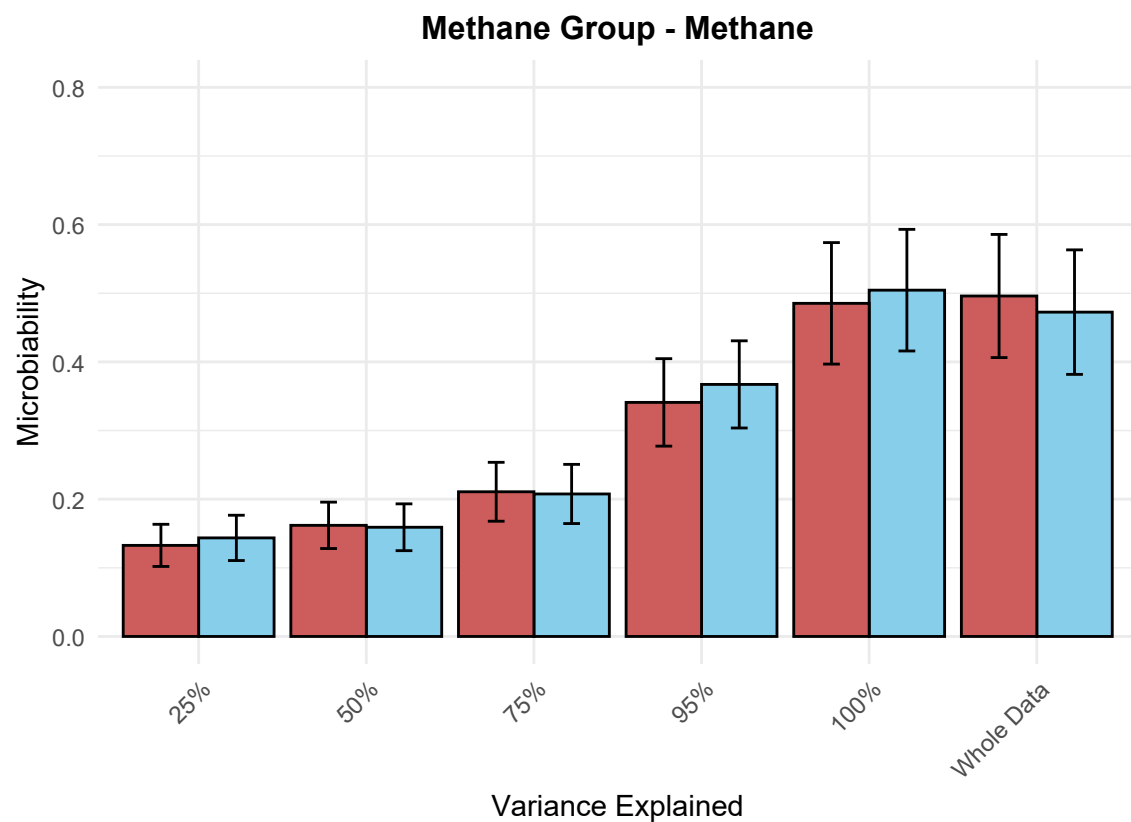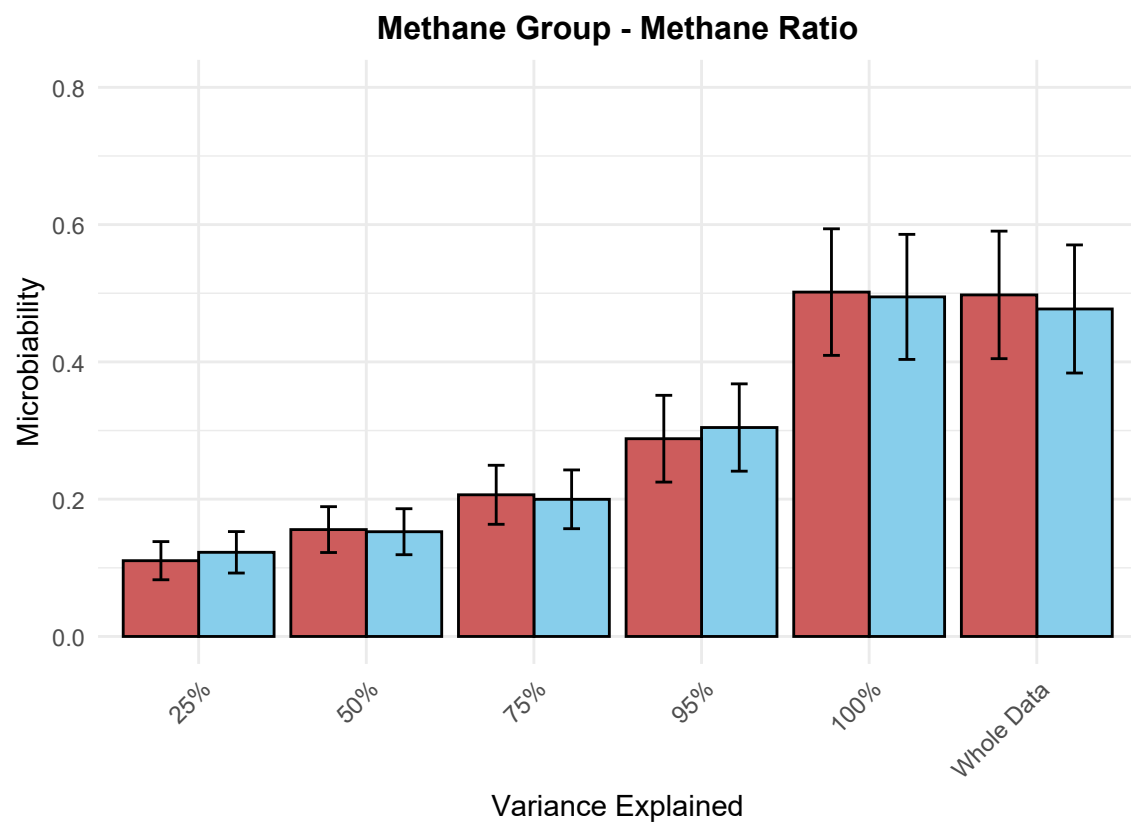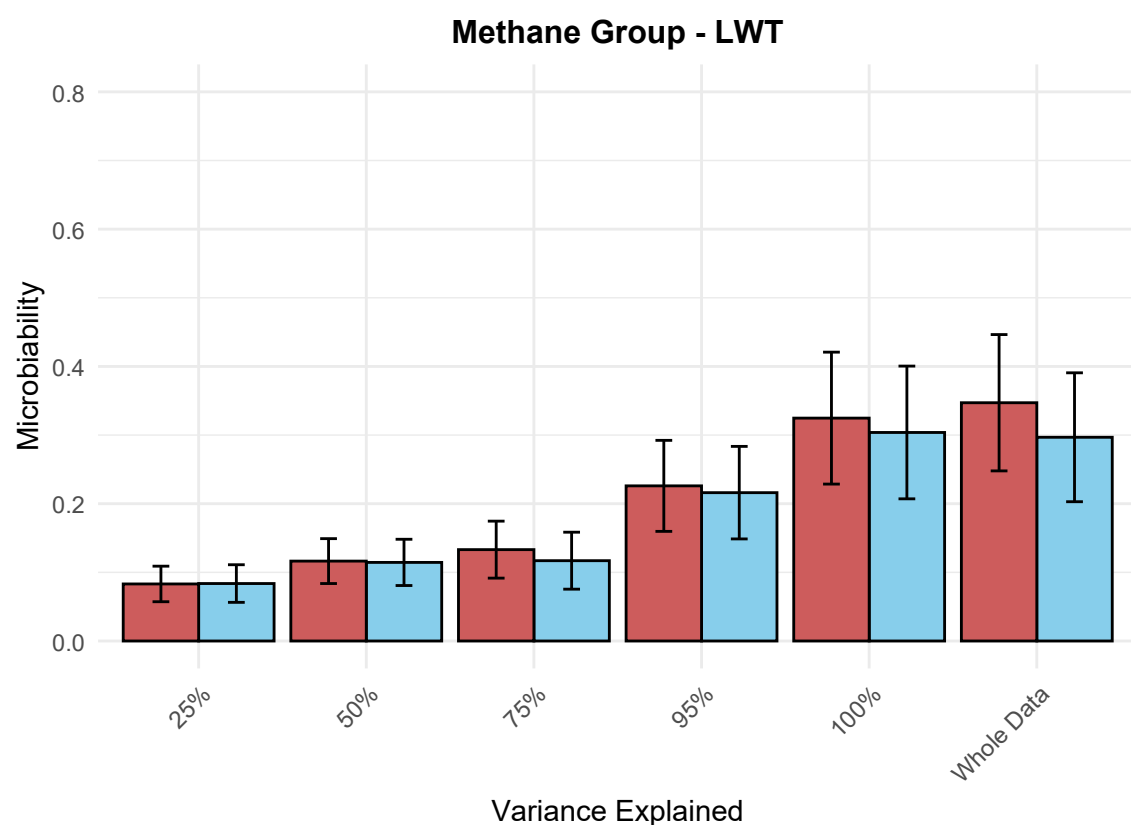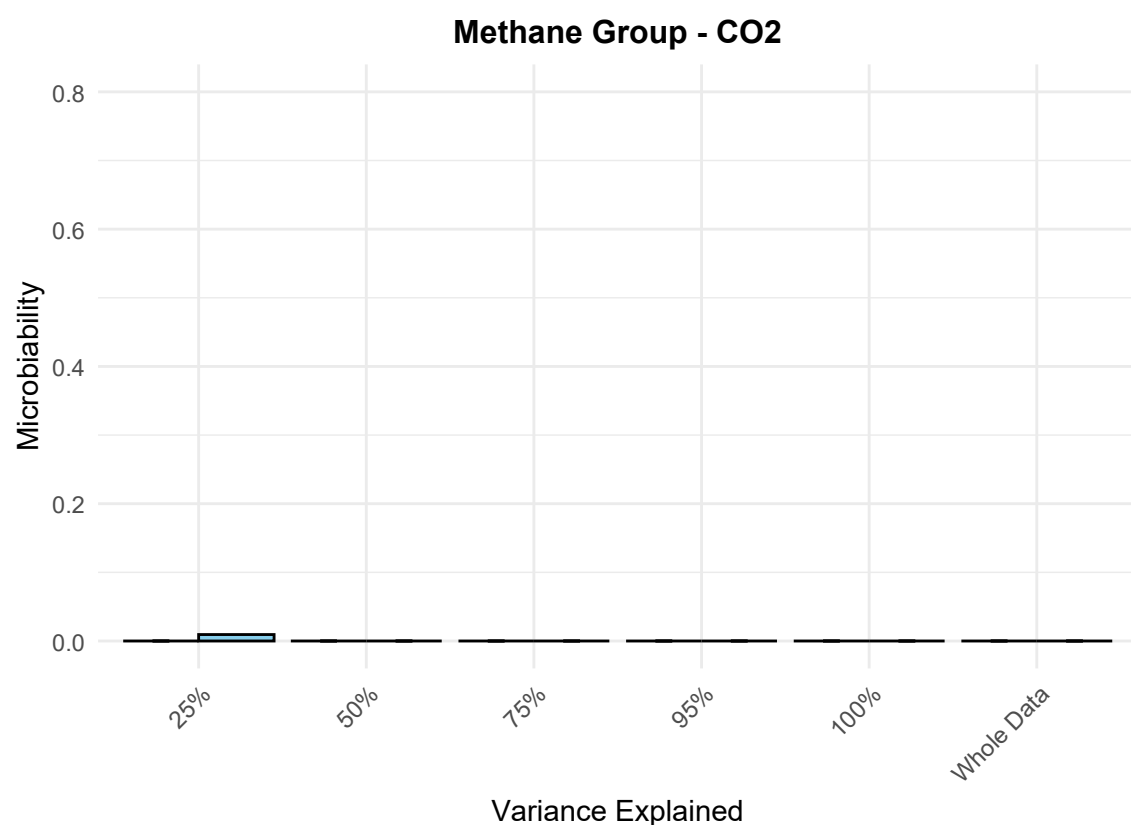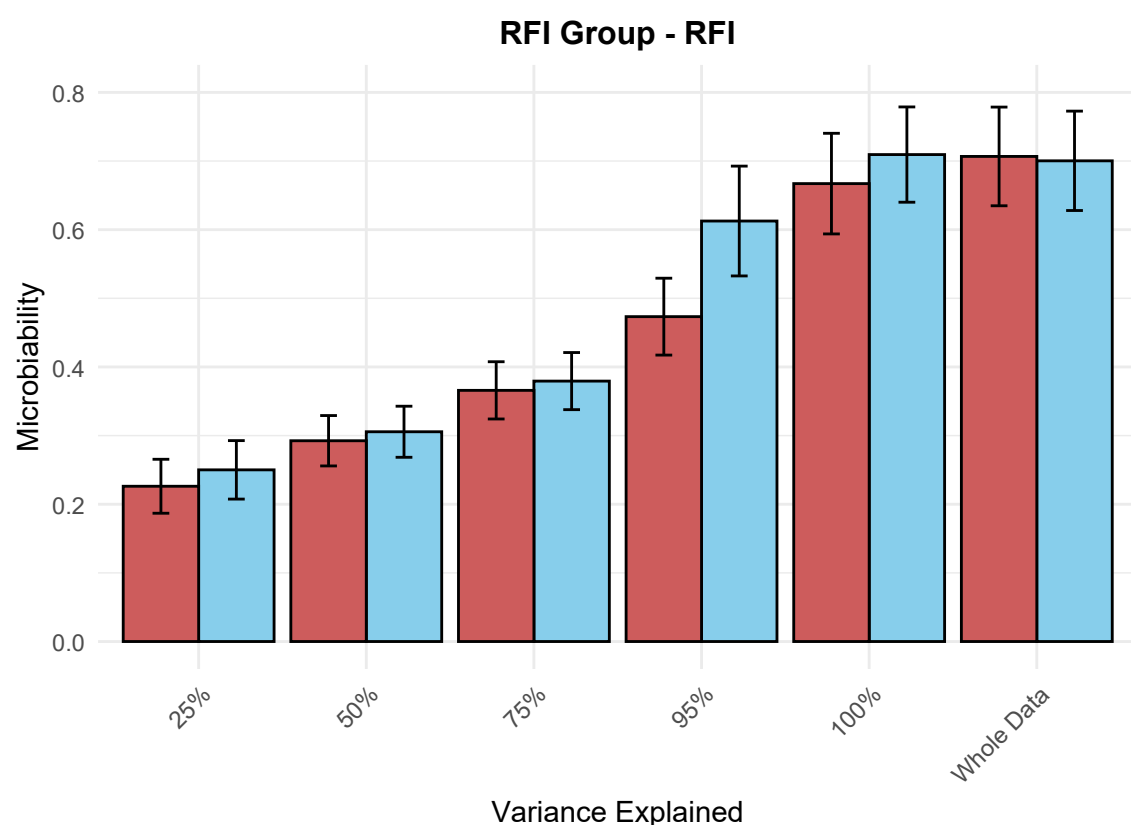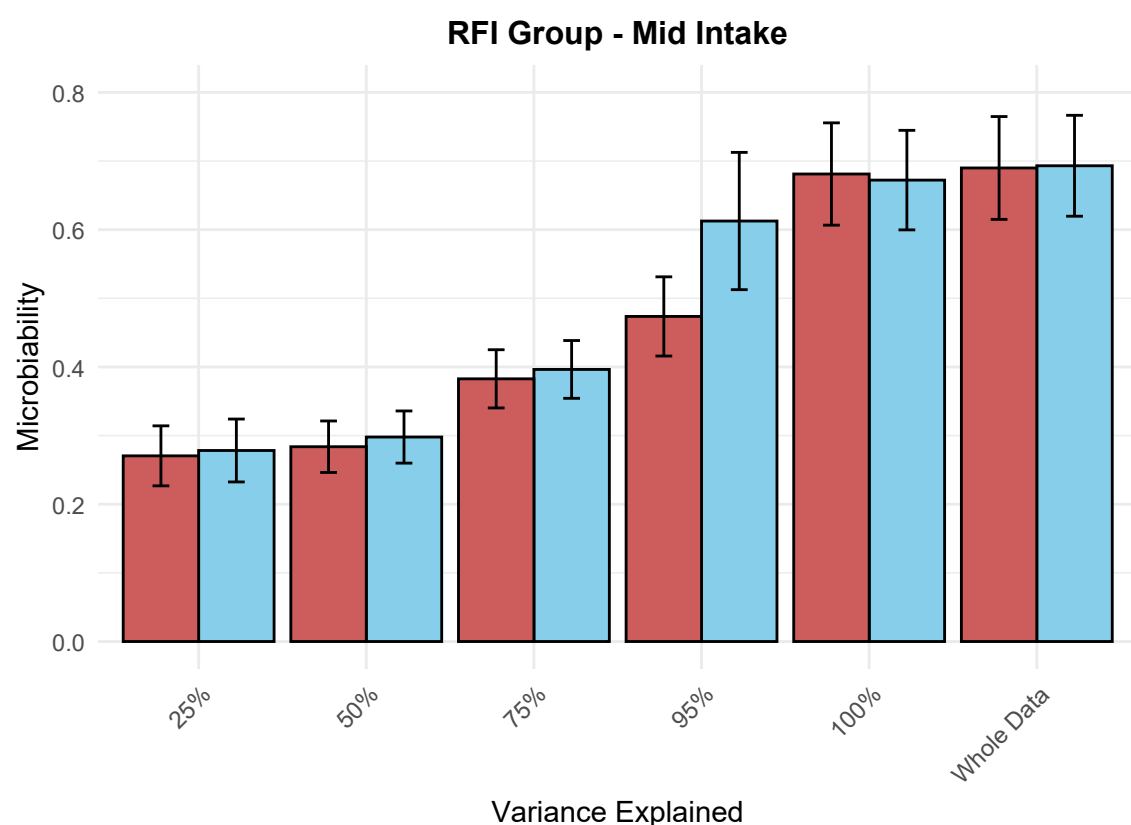

Supplement: Supplementary file 9 — Additional file 9. Comparison of Microbiability Estimates Using Log and CLR Transformations of Rumen Microbial Profiles across Different Variance Thresholds. [file 12711_2025_987_MOESM9_ESM.pdf]

## RFI Group: Genomic Prediction Results

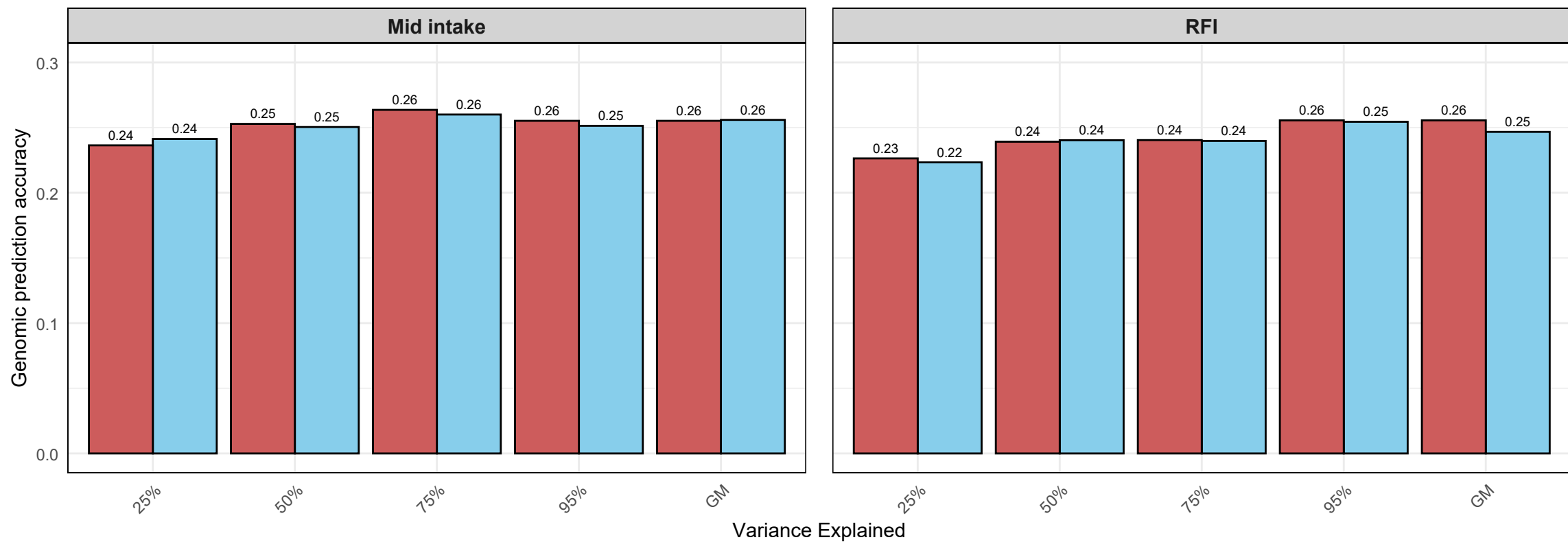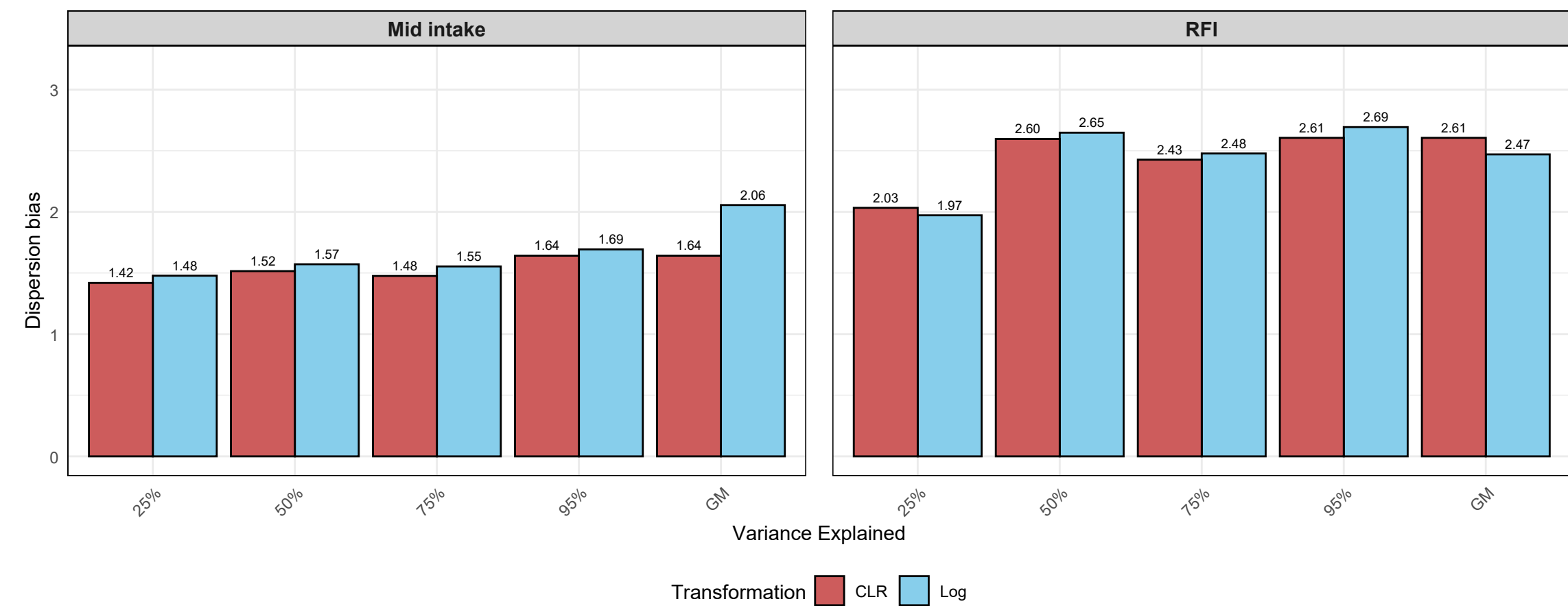

Supplement: Supplementary file 11 — Additional file 11. Genomic Prediction Accuracy and Bias for RFI Group Traits Using CLR and Log Transformations with the GM Model. [file 12711_2025_987_MOESM11_ESM.pdf]

**NNGBLUP Accuracy Comparison: Log vs CLR Transformation (25% and 50% Variance)**

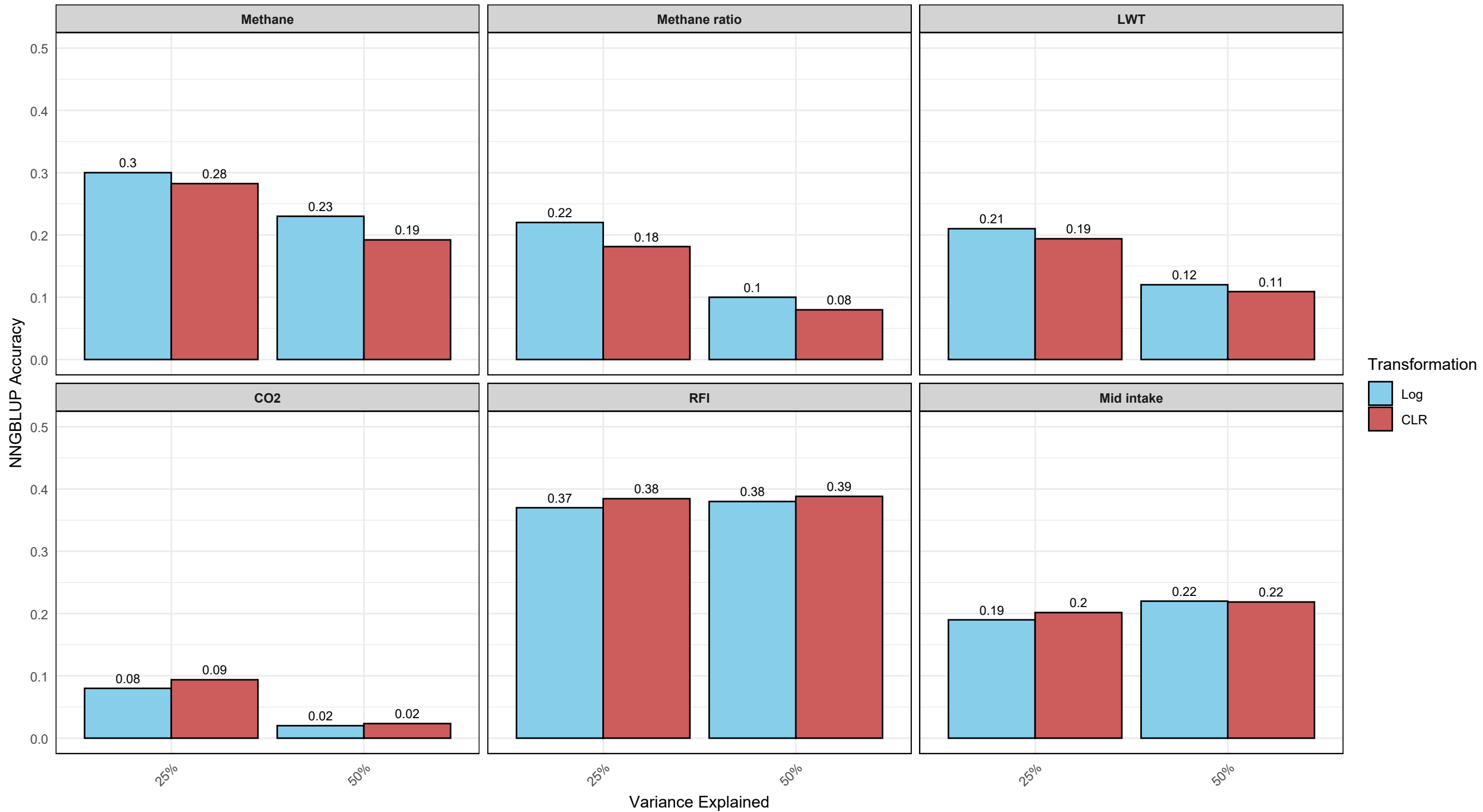

Supplement: Supplementary file 12 — Additional file 12. Comparison of NN-GBLUP Genomic Prediction Accuracy Using CLR versus Log Transformation across All Traits. [file 12711_2025_987_MOESM12_ESM.pdf]

**NNGBLUP Bias Comparison: Log vs CLR Transformation (25% and 50% Variance)**

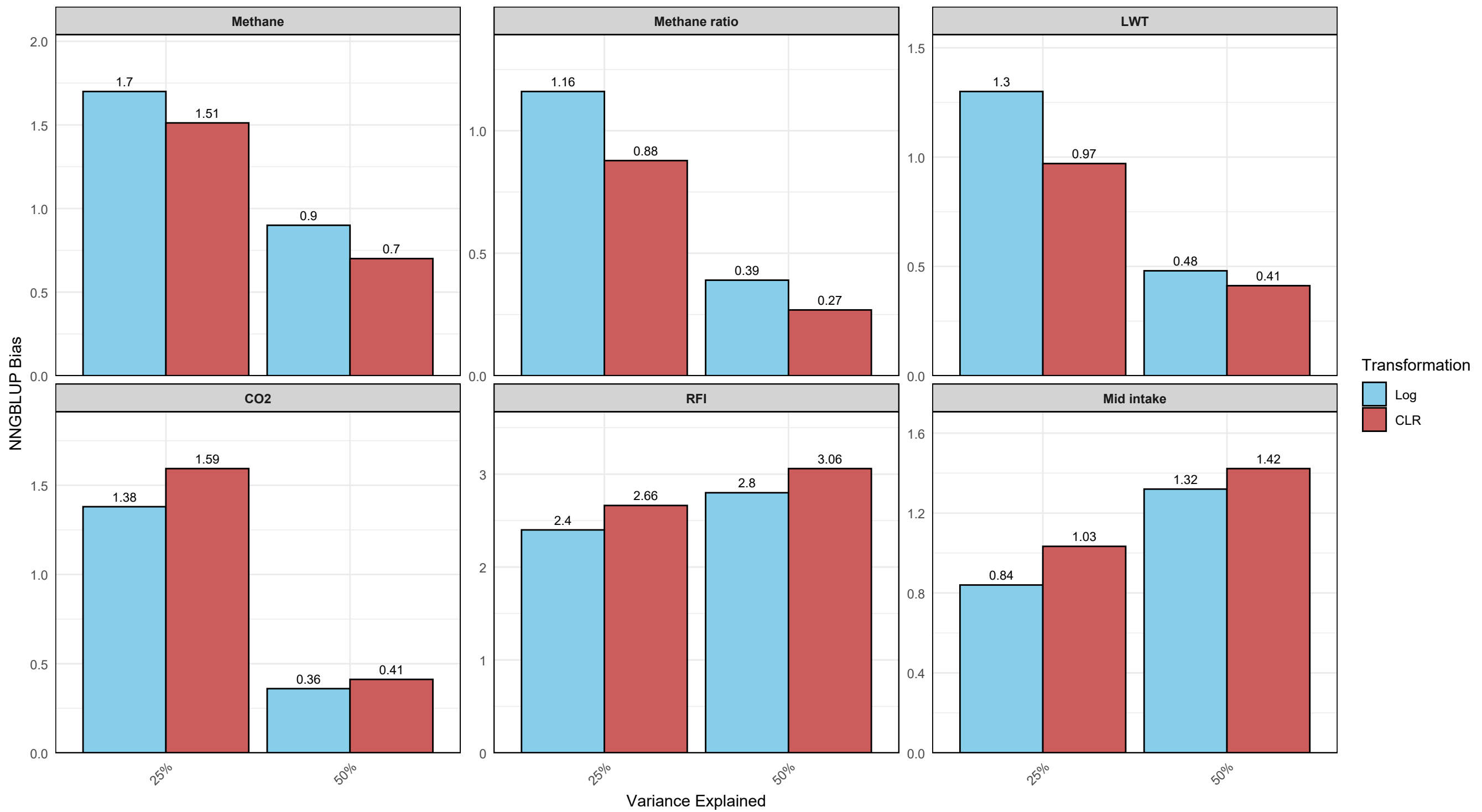

Supplement: Supplementary file 13 — Additional file 13. Comparison of NN-GBLUP Dispersion Bias Using CLR versus Log Transformation across All Traits. [file 12711_2025_987_MOESM13_ESM.pdf]
