# Supplementary material for: Improving genomic prediction accuracy for methane emission and feed efficiency in sheep: integrating rumen microbial PCA with host genomic variation using neural network GBLUP (NN-GBLUP)
Source: Genet Sel Evol. 2025 Jul 17;57:41. doi: 10.1186/s12711-025-00987-x (PMC12273308; doi:10.1186/s12711-025-00987-x)
Supplement: Supplementary file 8 — Additional file 8. Comparison of Cumulative Variance Explained by Principal Components Using Log and CLR Transformations of Rumen Microbial Profiles. [file 12711_2025_987_MOESM8_ESM.pdf]

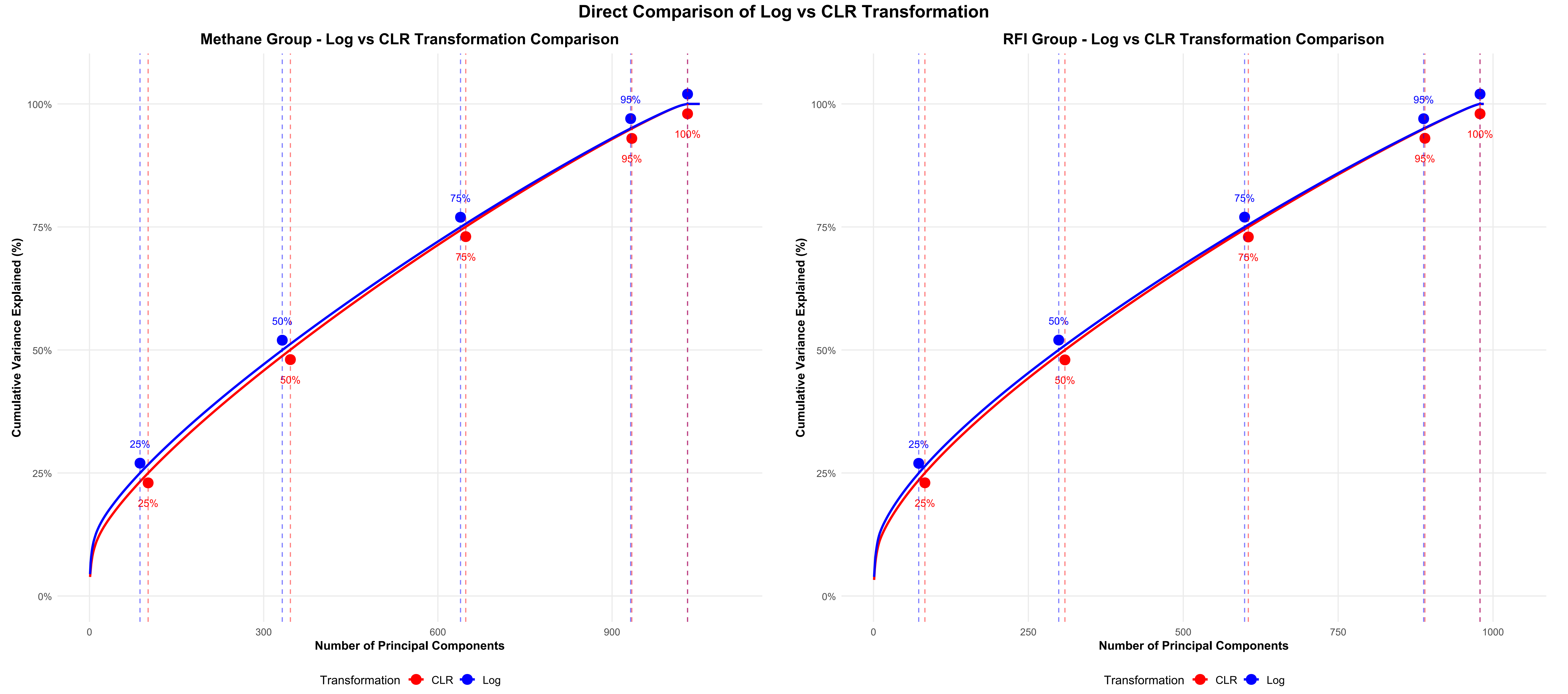
Direct Comparison of Log vs CLR Transformation
Methane Group - Log vs CLR Transformation Comparison
RFI Group - Log vs CLR Transformation Comparison
Cumulative Variance Explained (%)
Number of Principal Components
0%
25%
50%
75%
100%
0
300
600
900
250
500
750
1000
Transformation
CLR
Log
