# Supplementary material for: Improving genomic prediction accuracy for methane emission and feed efficiency in sheep: integrating rumen microbial PCA with host genomic variation using neural network GBLUP (NN-GBLUP)
Source: Genet Sel Evol. 2025 Jul 17;57:41. doi: 10.1186/s12711-025-00987-x (PMC12273308; doi:10.1186/s12711-025-00987-x)
Supplement: Supplementary file 10 — Additional file 10. Genomic Prediction Accuracy and Bias for Methane Group Traits Using CLR and Log Transformations with the GM Model. [file 12711_2025_987_MOESM10_ESM.pdf]

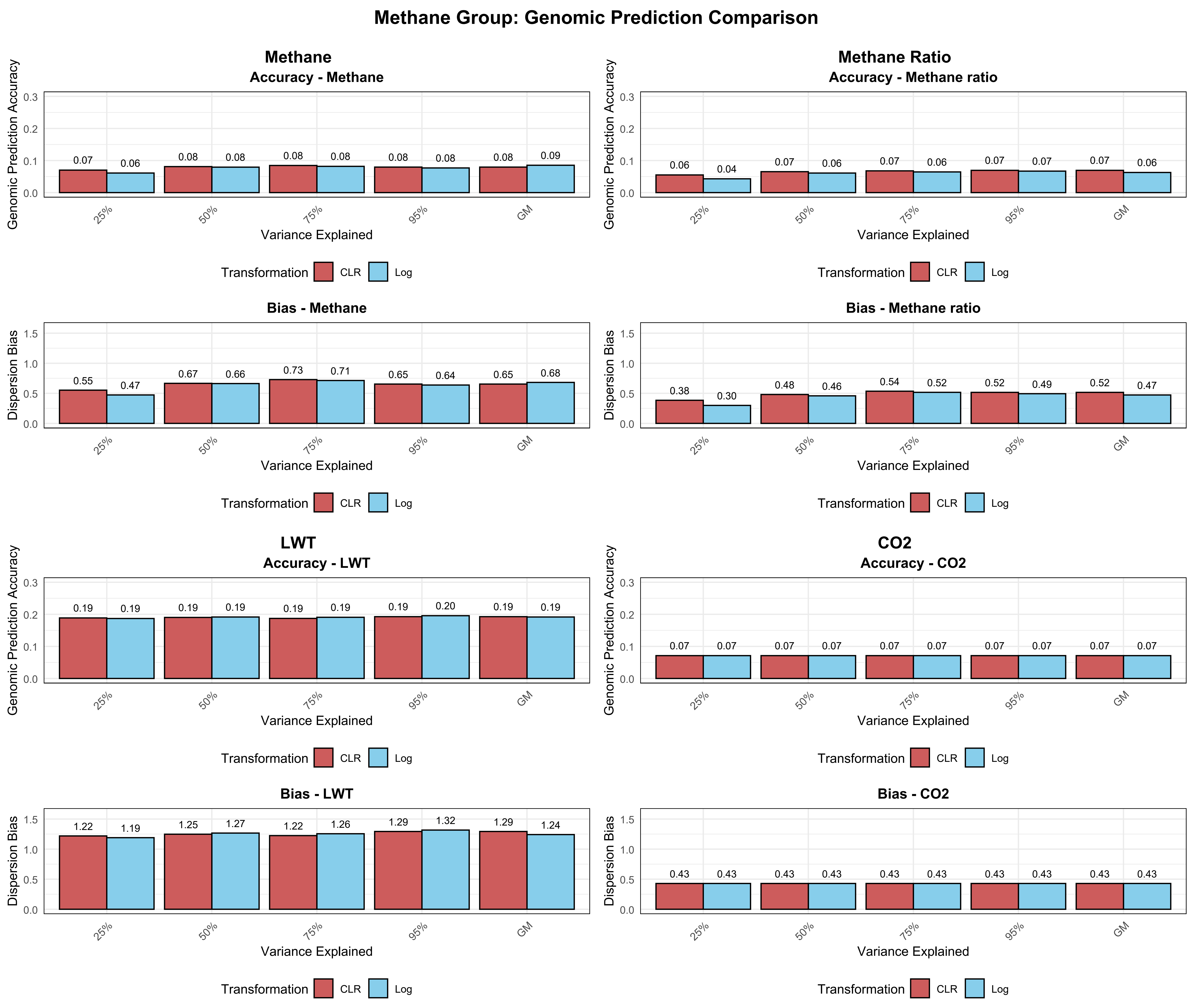
Methane Group: Genomic Prediction Comparison
Methane
Accuracy - Methane
Genomic Prediction Accuracy
0.07 0.06 0.08 0.08 0.08 0.08 0.08 0.08 0.08 0.09
25% 50% 75% 95% GM
Variance Explained
Transformation CLR Log
Bias - Methane
Dispersion Bias
0.55 0.47 0.67 0.66 0.73 0.71 0.65 0.64 0.65 0.68
Methane Ratio
Accuracy - Methane ratio
0.06 0.04 0.07 0.06 0.07 0.06 0.07 0.07 0.07 0.06
Bias - Methane ratio
0.38 0.30 0.48 0.46 0.54 0.52 0.52 0.49 0.52 0.47
LWT
Accuracy - LWT
0.19 0.19 0.19 0.19 0.19 0.19 0.19 0.20 0.19 0.19
Bias - LWT
1.22 1.19 1.25 1.27 1.22 1.26 1.29 1.32 1.29 1.24
CO2
Accuracy - CO2
0.07 0.07 0.07 0.07 0.07 0.07 0.07 0.07 0.07 0.07
Bias - CO2
0.43 0.43 0.43 0.43 0.43 0.43 0.43 0.43 0.43 0.43
